# Interhemispheric integration in visual search

**DOI:** 10.1016/j.neuropsychologia.2011.05.011

**Published:** 2011-07

**Authors:** Stewart Shipp

**Affiliations:** Department of Visual Neuroscience, UCL Institute of Ophthalmology, 11-43 Bath Street, London EC1V 9EL, UK

**Keywords:** Guided search, Bilateral field advantage, Spatial attention, Intrahemispheric competition, Ipsilateral representation, Inferior hemifield

## Abstract

The search task of Luck, Hillyard, Mangun and Gazzaniga (1989) was optimised to test for the presence of a bilateral field advantage in the visual search capabilities of normal subjects. The modified design used geometrically regular arrays of 2, 4 or 8 items restricted to hemifields delineated by the vertical or horizontal meridian; the target, if present, appeared at one of two fixed positions per quadrant at an eccentricity of 11 deg. Group and individual performance data were analysed in terms of the slope of response time against display-size functions (‘RT slope’). Averaging performance across all conditions save display mode (bilateral vs. unilateral) revealed a significant bilateral advantage in the form of a 21% increase in apparent item scanning speed for target detection; in the absence of a target, bilateral displays gave a 5% increase in speed that was not significant. Factor analysis by ANOVA confirmed this main effect of display mode, and also revealed several higher order interactions with display geometry, indicating that the bilateral advantage was masked at certain target positions by a crowding-like effect.

In a numerical model of search efficiency (i.e. RT slope), bilateral advantage was parameterised by an interhemispheric ‘transfer factor’ (T) that governs the strength of the ipsilateral representation of distractors, and modifies the level of intrahemispheric competition with the target. The factor T was found to be higher in superior field than inferior field; this result held for the modelled data of each individual subject, as well as the group, representing a uniform tendency for the bilateral advantage to be more prominent in inferior field. In fact statistical analysis and modelling of search efficiency showed that the geometrical display factors (target polar and quadrantic location, and associated crowding effects) were all remarkably consistent across subjects. Greater variability was inferred within a fixed, decisional component of response time, with individual subjects capable of opposite hemifield biases.

The results are interpretable by a guided search model of spatial attention – a first, parallel stage guiding selection by a second, serial stage – with the proviso that the first stage is relatively insular within each hemisphere. The bilateral advantage in search efficiency can then be attributed to a relative gain in target weight within the initial parallel stage, owing to a reduction in distractor competition mediated specifically by intrahemispheric circuitry. In the absence of a target there is no effective guidance, and hence no basis for a bilateral advantage to enhance search efficiency; the equivalence of scanning speed for the two display modes (bilateral and unilateral) implies a unitary second-stage process mediated via efficient interhemispheric integration.

## Highlights

► All subjects showed a significant bilateral field advantage in a visual search task. ► The group mean scanning speed is 20% faster for bilateral vs. unilateral displays. ► Bilateral field advantage (BFA) is absent for the target-absent condition, implying. ► BFA reflects intrahemispheric competition in a 1st, parallel stage of guided search. ► Interhemispheric competition in the 2nd, serial stage of guided search.

## 1 Introduction

### 1.1 Two hemispheres – twin foci?

Each cerebral hemisphere largely replicates the circuitry found in the other, and many of the basic circuits have no interhemispheric component – reciprocal connections between cortex and thalamus, for instance, are almost entirely uncrossed. The resultant capacity for independent, parallel processing provides a significant gain in efficiency if a task is able to recruit both hemispheres (Banich, 1998). In the visual domain, one of the clearest demonstrations is the doubling in number of items that can be tracked in bilateral vs. unilateral presentations (Alvarez & Cavanagh, 2005) – an example of the ‘bilateral field advantage’.

Predicting in advance whether a task will benefit from bilateral presentation is not necessarily obvious. One of the first notable demonstrations predicted the opposite outcome – a unilateral advantage (Sereno & Kosslyn, 1991). The task required a same/different discrimination of L or T characters presented in the same or opposite hemifields, and it was reasoned that a comparison would be more efficiently mediated within a single hemifield, to escape the requirement for interhemispheric collation of form information across the corpus callosum. The outcome was that bilateral presentations produced shorter reaction times by 100–150 ms. In accounting for the outcome the authors mentioned that two stimuli appearing simultaneously in the same hemifield might engender either a competition for common processing structures or intrahemispheric inhibition (Sereno & Kosslyn, 1991). From the vantage of two decades of psychophysical and physiological progress, these two mechanisms can be seen as opposite sides of the same coin.

Several subsequent demonstrations of the bilateral field advantage in item discrimination or detection tasks have the following design elements in common: the locations for the two attended items are indicated in advance by central cues, and their presentation is accompanied by a field of distractors (Awh & Pashler, 2000; Chakravarthi & Cavanagh, 2009; Kraft et al., 2005, 2007; Mounts & Gavett, 2004; Reardon, Kelly, & Matthews, 2009). Pooling across these studies, it is reasoned that the effect of focal attention is to free the target items from interference, or inhibition by distractors (Awh & Pashler, 2000); or in the terms of the ‘biased competition’ model, attention allows neural representations of the target items to win a competition with rival distractor representations to access higher processing resources (Desimone, 1998; Mounts & Gavett, 2004). Competition is fiercer between items located within the same hemifield, to dominate the resources of the contralateral hemisphere; hence, when bilaterally arrayed items are processed by both hemispheres, more resources are available (Chakravarthi & Cavanagh, 2009; Kraft et al., 2005). In very general terms, therefore, the bilateral advantage pertains to attentional selection (Reardon et al., 2009), as the visual system finds it easier to organize twin foci of attention in separate hemispheres than in the same hemisphere.

### 1.2 Single focus of attention

The foregoing analysis is at odds with the more traditional concept of spatial attention acting like a spotlight, or zoom lens – i.e. having a single (if malleable) focus (Eriksen & St James, 1986; McCormick & Klein, 1990; Posner, Snyder, & Davidson, 1980). Whether the focus of attention can be effectively split remains a contentious issue. A recent review opines that the “jury is still out” and offers the rationale that splitting the focus of attention is a strategy that subjects may adopt when so obliged by the constraints of the task confronting them (and one that benefits from training), but is not the default mode of operation of the attentional system (Jans, Peters, & De Weerd, 2010).

The rationale for a single focus gains support from the premotor theory of attention (Awh, Armstrong, & Moore, 2006; Corbetta, 1998; Moore, Armstrong, & Fallah, 2003; Rizzolatti, Riggio, Dascola, & Umilta, 1987) – the idea that the focus of covert attention is directed by the same mechanisms responsible for guiding serial fixations: clearly, the eyes do not fixate two targets at once. A covert search task can be pictured as a serial inspection of array items, and it is interesting that search is one attentional task where a bilateral advantage has been reported to be absent (Luck, Hillyard, Mangun, & Gazzaniga, 1989; Luck, Hillyard, Mangun, & Gazzaniga, 1994). This, however, is a result reported for the control group; the primary aim of these studies was to compare search speed for unilateral and bilateral arrays in a set of subjects who had undergone total or partial forebain commissurotomy. The result was that the split-brain subjects were able to scan bilateral displays twice as quickly as unilateral displays – as if, in this case, two spotlights were in simultaneous operation.

### 1.3 Twin foci in split-brain subjects

As this work is the basis for the study conducted here, it will be reviewed in more detail. Luck et al. (1994) report a group of 4 subjects with total (or near total) callosectomies and a control group, tested with a difficult search task in which the distractors were 180° rotated copies of a target formed of abutting red and blue squares. 2, 4, or 8 display items in total were equally distributed either side of fixation, or to the left or right side alone. Estimating the inspection time per item (ITI) by the slope of the response time functions in pooled group data, the commissurotomy subjects produced ITIs of 27.8 and 49.2 ms respectively for bilateral (BIL) and unilateral (UNL) displays, compared to 39.4 and 44.7 ms for the control group. The four commissurotomy subjects all performed similarly to each other, with UNL:BIL ratios varying from 1.5:1 to 2.0:1 – a rare demonstration of supranormal performance on the part of brain-damaged subjects.

The current report describes a (modified) replication of this study in a group of six normal subjects. Although the control group in the Luck et al. (1989, 1994) studies showed a mild but insignificant speed advantage for bilateral displays, it may be reasoned that a bilateral advantage should also be observable in search performed by normal subjects, given what is known of brain circuitry. In principle, a bilateral array should better utilise the resources of both hemispheres (Banich, 1998) – and being a detection, not a comparison task, it should not incur the penalty of interhemispheric collation. The argument hinges upon models of search as two-stage process, with a parallel process preceding, or complementing, a serial process (Muller-Plath & Pollmann, 2003; Treisman & Sato, 1990; Wolfe, 1994). The parallel stage acts to guide serial deployment of an attentional focus to improve on the efficiency of a random search, and it is envisaged to integrate bottom-up (salience) and top down (target feature) criteria to establish high likelihood target locations (Wolfe, 1994). Corticothalamic interactions are likely to form an important component of the relevant neural processing at the parallel stage, and these interactions are not bilaterally integrated (Robinson & Petersen, 1992; Sherman, 2005; Shipp, 2003, 2004; Snow, Allen, Rafal, & Humphreys, 2009). Hence there should be some gain in efficiency if the search items are not all processed by one hemisphere, but distributed between them, as this will place fewer distractors in direct competition with the target item.

The magnitude of the predicted advantage is awkward to forecast. The fact that bilateral displays enabled a doubling of search speed in split-brain subjects (Luck et al., 1989, 1994) can be ascribed to two factors: (i) the severance of forebrain interhemispheric links may have allowed complete operational independence of each hemisphere's ‘premotor’ (command) apparatus for deploying an attentional focus; (ii) the ipsilateral representation of items in each hemisphere was abolished, hence a unilateral array would provide one hemisphere with precisely twice as many items to search (and the other hemisphere would make no contribution).

In intact subjects the command system (i.e. the second, serial stage of a guided search model) may be fully bilaterally integrated, or it may have a degree of hemispheric autonomy, as registered in the split attention debate, above. Thus the role of factor (i) in intact subjects is equivocal. Regarding (ii), the nature of the ipsilateral representation in nonhuman primate visual cortex is known from maps of commissural connections (Clarke & Miklossy, 1990; Demeter, Rosene, & Van Hoesen, 1990; Van Essen, Newsome, & Bixby, 1982; Van Essen & Zeki, 1978), allied to neurophysiological receptive field mapping studies in certain areas, and confirmed by human fMRI studies (Tootell, Mendola, Hadjikhani, Liu, & Dale, 1998). These show that ipsilateral representation is limited to fringe regions adjacent to the vertical meridian in the early retinotopic areas, but progressively expands in the subsequent chain of form processing areas, with less orderly retinotopy, located in ventral occipital human cortex. In lateral occipital and fusiform visual areas, for instance, the gross ipsilateral response (as measured by BOLD signal in fMRI) is about half to two thirds as powerful as the contralateral response (Hemond, Kanwisher, & Op de Beeck, 2007). These observations imply that, in the context of the current hypothesis, bilateral advantage in search could be contingent on the nature of the target/distractor discrimination, and would be greater if the guidance for target location were to arise from relatively early areas where ipsilateral representation is minimal. The nature of the target/distractor discrimination required by the search task used by Luck and colleagues may not be the most appropriate, in this context – but this is not a clear assessment, and the same basic task was retained for the current study for the sake of comparability.

### 1.4 Design modifications

Some aspects of the experiment were redesigned to optimise sensitivity of the slope of the response function to controlled display factors, including regularisation of the geometry of the search arrays, and modification of the response procedure. Search items in Luck et al. (1989, 1994) appeared at random locations within fixed windows either side of fixation; hence item density was twice as great in the unilateral displays which, as the authors acknowledged, may have led to greater lateral inhibition. In the current design there were 8 fixed positions (i.e. 2 per quadrant) shared by target and distractors, and 8 additional positions for distractors alone. The requirement for equal density led to a quadrant based geometry, with bilateral displays occupying both superior, or both inferior quadrants. This design not only eliminated unequal density, but also added the capability to examine effects linked to display elevation: attentional resolution is known to be coarser in superior field (He, Cavanagh, & Intriligator, 1996; Intriligator & Cavanagh, 2001), and several studies have noted field asymmetries in visual search (Efron & Yund, 1996; Previc & Blume, 1993; Previc & Naegele, 2001; Yund, Efron, & Nichols, 1990a) or in bilateral advantage paradigms (Awh & Pashler, 2000; Carlson, Alvarez, & Cavanagh, 2007; Kraft et al., 2005; Mounts & Gavett, 2004).

Subjects were instructed to report the quadrant of location of the target, but only as the second phase of a 2-step report. The first step was the timed response, a single-key press that triggered replacement of the display items by masking items at all 16 positions. The response procedure in Luck et al. (1989, 1994) was a right- or left-thumb button press to indicate right or left target location. The requirement for response selection would be expected to extend the response time, and may have done so in a differential fashion if the decision was more taxing in respect of bilateral displays.

The final modification to the procedure of Luck et al. (1989, 1994) was the inclusion of a target-absent condition. The simplest prediction for this condition, if subjects carry out an exhaustive serial search, is that the slope of the response function will double. The prediction arising from parallel or guided-search models is less succinct. The absence of a target means that any ‘guidance’ simply reflects internal noise, that subjects may not search exhaustively; they might inspect items down to a certain threshold activation above background, or simply give up when a suitable time period without success has elapsed (Chun & Wolfe, 1996). However, the absence of a target does preclude any possible bilateral advantage arising from a more efficient guidance stage. Hence, were the target-absent condition to show a bilateral advantage, it could only be ascribed to attentional selection along the same lines as the previous demonstrations, nominally contingent on some degree of hemispheric autonomy in the second stage of a guided-search process.

## 2 Methods

### 2.1 Subjects and stimulus design

**Fig. 1:**
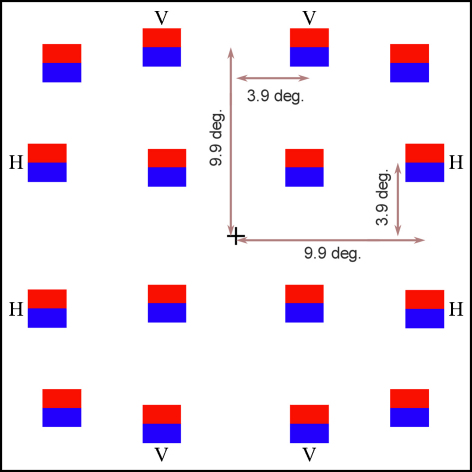
Item locations in the search task. There were two target locations per quadrant, labelled V and H, centred at 3.9° and 9.9° from the vertical and horizontal meridians (or vice versa). The remaining eight locations were distractor-only locations, centred on the main diagonal at 3.6 or 9.1° from each meridian. Hence V and H target loci were at an eccentricity of 10.6°, the near distractor was at 5.1° and the far distractor at 12.7°. The target, if present, was a single reversed distractor (i.e. blue-above-red). Items occupy a pair of adjacent quadrants in all displays: 1 V- and 1 H-locus in 2-item trials, 2 V- and 2 H-loci in 4-item trials, and all 8 available positions in 8-item trials. (For interpretation of the references to colour in this figure legend, the reader is referred to the web version of the article.)

Six college students (3 males) all aged 20–24 gave informed consent for this experiment, in accord with UCL ethical procedures for human subjects. All were right handed, with an average laterality index of 88.1 as scored by the Edinburgh Handedness Inventory (Oldfield, 1971). Subjects viewed a monitor (Sony multiscan 20se II) at a distance of 60 cm from a fixed chin support. Visual stimuli were generated by Cogent Graphics (developed by J. Romaya at LON, UCL: http://www.vislab.ucl.ac.uk/cogent.php). The displays were composed of 2, 4 or 8 stimulus items, in a regular geometric array (Fig. 1). The items were 2° × 2° in size, presented at fixed positions centred a maximum 12.7° and a minimum 5.1° from a fixation cross. Target items were at an eccentricity of 10.6°. The minimum spacing (in the 8-item displays) was 5.2° (i.e. a minimal gap of 3.2° between the nearest edges). The regular geometric design equalized any crowding effects across quadrants. Each item was square shaped, with the upper and lower halves differing in colour; target items were blue above red, distractor items were red above blue. The luminance of the blue component was set to 6.1 cd/m^2^, and the red component was individually adjusted to isoluminance using a minimum flicker method. The grey background was of luminance 0.6 cd/m^2^.

Stimulus items were presented in two adjacent quadrants, with 2, 4 or 8 items located symmetrically about either the vertical or horizontal meridian (or with rotational symmetry in the case of the 2-item display). This gave four basic configurations: bilateral inferior, bilateral superior, unilateral right and unilateral left (BI, BS, UR and UL). Target items were presented at two possible locations per quadrant, centred near (3.9°) to either the vertical or horizontal meridian (respectively, the ‘V-locus’ and ‘H-locus’). Targets appeared with equal frequency across the eight available positions, and the frequency of target-absent trials was fixed at 20%. Hence a single, pseudo-randomised block of 60 trials contained 2-, 4- and 8-item variants of the BI, BS, UR and UL configurations, each appearing with a target at one of its four possible locations, or in target-absent mode.

### 2.2 Procedure

Subjects commenced each trial with a blank screen plus fixation cross. Eye position was monitored with an ASL 504 tracking system and eye movements exceeding 1.8° from fixation terminated the trial, with a warning message to the subject. The stimulus array appeared after a randomly varied period of 1.0–1.7 s fixation. Subjects were instructed to detect a target item and to identify its quadrantic location, or to decide that no target was present. The detection/decision time was recorded by an initial key press (mouse button). Subjects then moved the mouse to the appropriate quadrant to indicate target location. On the first key press, display items were masked by a red/blue checkerboard pattern (200 ms) before vanishing, to prevent any contamination of the subject's location response by iconic visual memory. Target-absent decisions were indicated by a mouse click at the fixation point. No feedback was given regarding correct or incorrect responses.

All subjects had training sessions (of variable duration) until they were performing at 85% correct, and then completed 3 experimental sessions. A single session comprised 8 blocks of (minimally) 60 trials (i.e. 480 in total). Blocks typically contained in excess of 60 trials, because trials with incorrect responses were repeated toward the end of the block until a correct response was obtained. Trials marked ‘incorrect’ included those with response times outside the range 0.3–2.5 s, in addition to trials where the subject's response was incorrect with regard to target presence, or location. The session was punctuated by seven pauses (i.e. one after every 60 correct trials) at which point subjects were instructed to switch the hand used for responding (to counterbalance any effect of handedness on right/left asymmetries in response times).

### 2.3 Statistical data analysis

The aim was to establish the main effects (and interactions) of the experimental factors upon the slopes of response time functions (‘RT slopes’) obtained by regression statistics. Given the susceptibility of regression to outliers, the data were first filtered at ±2 SD (approx.) from the mean; the filter was applied at the lowest possible level, i.e. to the block of 12 observations representing a unique combination of factors for each subject. The cut-off point was initially set at ±2 SD, then adjusted within a range 2.1–2.4 SD to standardize the reject rate at 5% across subjects.

For group data analysis the RT slopes were submitted to a 5-way ANOVA, with subject declared as a random factor. The experimental variables were one display mode factor (unilateral, bilateral), three target position factors – ‘locus’ (V or H), ‘elevation’ (superior, inferior) and ‘laterality’ (left, right) – and the response factor ‘hand’ (left, right). Because the factors were modelled as having a multiplicative effect upon RT slope, the slope data for all ANOVAs were log-transformed. The target-absent RT slope data were submitted to a separate 2-way ANOVA with the factors display and hand (i.e. lacking the three target location factors).

Individual performance data was analysed by regression statistics and ANCOVA based on the response times recorded in individual trials. Three different regression models are compared: model A plots a single regression function for the entire dataset; model B plots two regression functions (e.g. for unilateral vs. bilateral display mode) with equal slope, but differing in intercept. Model C plots two functions with differing slope and intercept. The *F*-test for a significant difference in mean response time compares model B to A, and the *F*-test for a significant difference in RT slope compares model C to B.

### 2.4 Data modelling

An optimisation routine (the ‘solver’ function in Excel) was used to obtain exact solutions for a factorial model of RT slope data, specifying the three factors D (display), E (elevation) and L (locus), each with binary modes (respectively, U/B = uni/bilateral; S/I = sup/inferior; V/H = vertical/horizontal target locus). The modelled data were the eight RT slopes corresponding to each unique combination of these factors (after pooling data with regard to the factors laterality and hand) – USV, USH, UIV, UIH, BSV, BSH, BIV, and BIH. The model had eight variables: a base value for RT slope (or ‘base speed’) *x*_0_, and seven further variables (*x*_1_–*x*_7_) representing, respectively, the main effect of each factor, the three 2-way interactions, and the single 3-way interaction. In the initial set up, the weight of each factor effect was distributed equally between its component modes. For example, the main effect of factor D has modes *U* and *B*. One of these (say *B*) has a weight (*x*_1_) and *U* is yoked to *x*_1_: i.e. *B* = *x*_1_ and *U* = 1/*x*_1_. The magnitude of the factor is the ratio *U:B*, or *x*_1_^2^. For a second example, the interaction factor D*L has component modes *UV*, *UH*, *BV* and *BH*. Let *BV* = *UH* = *x*_5_: and *UV* = *BH* = 1/*x*_5_. Here, the total power (or magnitude) of the factor is *x*_5_^4^. The optimisation routine, when implemented, finds values of *x*_0_, *x*_1_…*x*_7_ to solve eight simultaneous equations, each matching an observed RT slope to the product of *x*_0_ with *x*_1_, *x*_2_…*x*_7_, or their reciprocals, as appropriate to the definition of each component mode; e.g.

$$\text{Observed}\;\text{RT}\;\text{slope}\;\text{USV}=x_{0}\times U\times S\times V\times US\times UV\times SV\times USV$$

The set of formulae for all eight RT slopes appear in Appendix A. Model development proceeds by re-assigning the weight distribution of a factor across its components. For example, factor D*L weights were modified to BV = UH = *x*_5a_, and UV = BH = 1. The magnitude of a factor effect, as determined by the optimisation routine, is not affected by reassigning its component weights – in the foregoing example *x*_5a_^2^ = *x*_5_^4^. However, the magnitude of a factor can be affected by weight reassignment in higher level factors. The weight reassignment within a factor can be made more complex; e.g. Section 3.4.6.1 outlines the rationale for allowing the component weight settings of one factor to depend on the values of another.

## 3 Results

### 3.1 Summary outcome

**Fig. 2:**
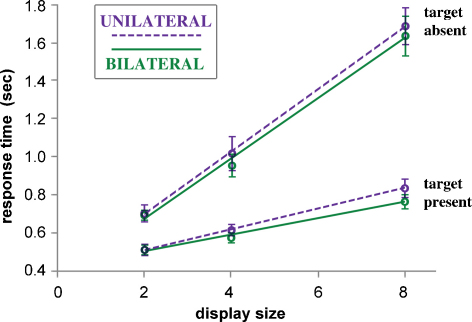
Response time plots for pooled subject data. The data are collapsed over the response factor ‘hand’, and over all variants of stimulus geometry save for display mode (unilateral vs. bilateral) and presence/absence of target. Statistical assessment of effect of display mode based on population of equivalent plots for individual subjects: target-present: *F*(1,5) = 10.3, *p* = 0.024; target-absent: *F*(1,5) = 0.83, *p* = 0.40). Error bars indicate ± s.e. of subject means.

The principal matter of enquiry is the effect of display mode (unilateral vs. bilateral) on processing time per display item, and the difference between target-present and target-absent conditions. In other words it is specifically the significance of changes in slope, in plots of response time against item number, that is of central concern. Fig. 2 shows the overall outcome of the study in this respect, pooling data across all six subjects and all possible target locations. When a target is present the search speed is significantly faster for bilateral displays (43 ms per item) vs. unilateral (54 ms per item). Conversely, when the target is absent, there is no significant difference (bilateral 156, unilateral 165 ms per item). The ratio of search speeds for target-absence and presence is greater than that commonly found in such studies, but is not atypical for a search task that is relatively difficult (Wolfe, 1998).

This overall result conceals major effects related to other display variables. Unexpectedly, the location of a target within a quadrant (i.e. closer to the vertical or horizontal meridian) proved to be a major determinant of response time. And this effect was itself dissimilar across superior and inferior quadrants. In order to systematize these effects and analyse inter-subject variations, the RT slopes were parameterised in terms of target locus and quadrant-specific processing factors (describing variations in basic processing speed, its modulation by inferred crowding effects, and the nature of the representation of ipsilateral display items). The model arises from the factorial analysis implemented by ANOVA, considered first.

### 3.2 Factor analysis by ANOVA

Of the six experimental variables, five related to the display organisation, and one was a response variable (‘hand’). The geometry of the display is described by display mode (‘display’ – unilateral or bilateral), and item number (2, 4 or 8). The latter, of course, is used as the independent variable in the regression plot of response time, to form a new dependent variable, RT slope. Target location within the display is described by ‘locus’ (near to VM or HM within a quadrant), ‘elevation’ (superior or inferior), and ‘laterality’ (left or right). Note that (as implicit in factor analysis) a ‘right’ target location can refer to the right quadrant of a bilateral display, or the right hemifield presentation of a unilateral display; similarly, ‘superior’ locations include both bilateral and unilateral display geometries (with analogous ‘left’ and ‘inferior’ target locations).

The five binary factors yield 32 different conditions and associated RT slopes, each a product of 36 RT observations per subject (prior to filtering). The 5-way ANOVA, compiled from 192 estimates of RT slope across six subjects, therefore assesses the effect of each factor, and factor interactions, against the yardstick of intersubject variability. The outcome revealed significant effects related to display, locus and elevation; the factor laterality lacked significance as a main effect and as any form of interaction, and there was a solitary interaction involving hand. The factor display was significant as a main effect [*F*(1,5) = 34.2, *p* = 0.0021] and in the following interactions: display*locus [*F*(1,5) = 72.1, *p* = 0.00037], display*elevation [*F*(1,5) = 12.9, *p* = 0.016], display*locus*elevation [*F*(1,5) = 6.7, *p* = 0.048] and display*locus*hand [*F*(1,5) = 8.5, *p* = 0.033]. Other effects were governed by target position independent of display mode. There was a main effect of locus [*F*(1,5) = 8.5, *p* = 0.033] and a stronger interaction for locus*elevation [*F*(1,5) = 42.5, *p* = 0.0013]. There was no main effect of elevation per se [*F*(1,5) < 0.1].

### 3.3 The assessment of bilateral advantage

**Fig. 3:**
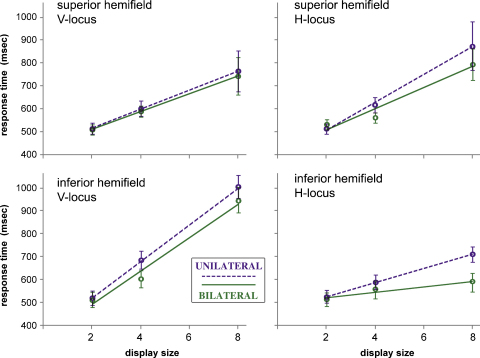
Response time plots for pooled subject data at specific target positions. These plots illustrate group performance for targets presented at the V- or H-locus, in superior or inferior hemifield (averaged across right and left target hemifield and response hand). Statistical assessment of effect of display mode (unilateral vs. bilateral) based on population of equivalent plots for individual subjects: superior V-locus, *F*(1,5) < 0.1, *p* > 0.5; superior H-locus, *F*(1,5) = 1.1, *p* = 0.33; inferior V-locus, *F*(1,5) = 1.5, *p* = 0.28; inferior H-locus, *F*(1,5) = 35.6, *p* = 0.0019. Error bars indicate ± s.e. of subject means.

Because the location of a target to the right or left of a display has no systematic effect upon response time, the effect of bilateral vs. unilateral display mode can be examined at four generic locations: the V or H loci in superior and inferior hemifields. Fig. 3 shows the RT functions compiled from pooled group data for the four target locations. The bilateral advantage in search speed is most apparent at the H locus, and is considerably greater in the inferior hemifield: the UNL:BIL ratios of RT slope at the H-locus are 2.61 and 1.37 in inferior and superior hemifield, respectively. For targets at the V locus, the corresponding ratios are 1.09 and 1.07.

**Fig. 4:**
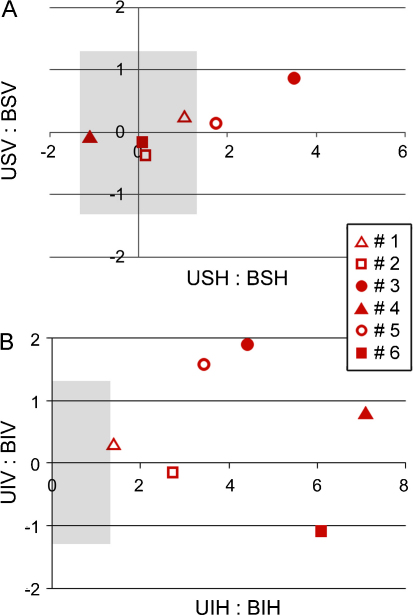
Individual variation in subjects #1 to #6 of the bilateral advantage across four target positions (superior and inferior V-locus and H-locus). Data plotted are the negative log transformations of *p* values obtained by individual subject ANCOVAs, each pertaining to the significance of the difference in RT slope under bilateral and unilateral display modes. (A) shows the superior quadrant data and (B) the inferior quadrant. Results for the H-locus are plotted horizontally and the V-locus vertically. The convention is adopted that the positive direction in each axis represents a bilateral advantage; the shaded areas enclose regions where −1.3 < −log *p* < 1.3 (i.e. *p* > 0.05) at both loci. Note that there are no instances of a significant unilateral advantage.

The magnitude of the bilateral advantage in pooled data reflects the consistency of the effect seen at each of these four target positions in the performance of individual subjects, as summarised by Fig. 4. Plots like those of Fig. 3 were constructed for each subject separately, and the difference in RT slope caused by display mode at each position assessed by ANCOVA. All subjects showed a notable enhancement of search speed with bilateral displays when the target was located at the inferior H locus. Two subjects (actually the same pair, #3 and #5) also showed a significant bilateral advantage for targets located at the superior H locus, and the inferior V locus, whilst display mode had no effect upon search speed for targets at the superior V locus.

### 3.4 A factorial model of target detection performance

The magnitude and direction of action of the three factors, locus, elevation and display, and their interactions, were obtained using an optimisation routine (the ‘solver’ function in Excel). This model treats the seven factor effects, *x*_1_–*x*_7_ (three main effects, three 2-way interactions, and one 3-way interaction) as multiplicative effects upon a base processing speed (*x*_0_). Hence the model uses eight parameters to construct the eight search speeds (as shown in Fig. 3). These parameters were obtained for the group data, and for each individual subject.

#### 3.4.1 Intersubject consistency

The magnitude of any given factor effect naturally varied from subject to subject, but all factors bar one were consistent in their direction of action. For instance all subjects showed a main effect of display favouring faster search speed in bilateral displays, and a main effect of locus favouring the H-locus. The only factor (or factor interaction) to vary in its direction of action across subjects was the main effect of elevation, that favoured superior target locations in three subjects, and inferior locations in the remainder. The chance probability that a factor acts consistently across all six subjects is 0.5^5^, or 0.031. The probability that as many as six out of seven (independent) factors give such an outcome is 6.3 × 10^−9^. By this measure, the influence of the display geometry and target location factors upon individual subject performance was remarkably consistent, and valid for modelling.

This initial estimation of factor magnitudes simply quantifies the influence of display geometry on search efficiency. The following sub-sections describe the development of a more ‘biological’ model, whose parameters were constructed by combining factors, and by re-assigning the weight distribution of a factor across its component modes. Both manipulations may be illustrated by the derivation of the first set of model parameters, the intrinsic processing speeds of the four target positions.

#### 3.4.2 Model parameters: (a) position-specific processing speeds

The source of variation in processing speed across target position is not known, but it can be treated as an inherent property of the relevant neural circuitry. These intrinsic processing speeds are obtained by combining the base speed (*x*_0_) with the relevant modes of three factors – the main effect of elevation, the main effect of locus, and the interaction factor elevation*locus. For example:

(1) $$\text{Intrinsic}\;\text{processing}\;\text{speed}\;\text{of}\;\text{the}\;\text{superior}\;\text{V}\;\text{locus}=x_{0}\times S\times V\times SV$$

Thus the intrinsic processing speed at each target position is formed by the product of *x*_0_ with the weights of the appropriate modes of the relevant factors.

##### 3.4.2.1 Weight reassignment within component modes of a factor

In the initial application of the optimisation routine, all component modes of a factor were assigned equal weights, or their reciprocals. For example, the factor elevation*locus has modes *SV* = superior vertical, *IH* = inferior horizontal, etc., weighted: SV = IH = IV^−1^ = SH^−1^. In the model of group data this gives:

(2) $$x_{6}=SV=IH=0.66\;\text{and}\;x_{6}^{-1}=IV=SH=0.66^{-1}$$

[NB: *x*_6_ = 0.66 is a speed increment, as it is an effect upon RT slope, i.e. processing time per item]. Now, even if the cause of the variation in processing speed with position is unknown, it is implausible to suppose that there are precisely equal and opposite influences acting upon each of the four positions – especially as the two H-loci are relatively nearby, and the two V-loci relatively far apart within the visual field. A more realistic supposition is to re-assign the weights as follows:

(3) $$x_{6}=SV=0.66^{2}\;\text{and}\;x_{6}^{-1}=IV=0.66^{-2}\;\text{and}\;IH=SH=1$$

The interaction is now depicted as a relative acceleration and deceleration of the two V-loci, in respect of a neutral effect upon the pair of H-loci. The adjustment of these weights brings on a compensating change in the weights of the main effect of elevation (with modes *S* = superior and *I* = inferior). For the group data:

(4) $$\begin{array}{l} \text{initially},\;x_{2}=I=0.91\;\text{and}\;x_{2}^{-1}=S=0.91^{-1}\\ \text{post}\;\text{adjustment},\;x_{2}=I=0.91\times 0.66\;\text{and}\;x_{2}^{-1}=S=0.91^{-1}\times 0.66^{-1} \end{array}$$

The change in elevation factor enhances the advantage of inferior target positions over superior positions, to counterbalance the weight reassignment within the elevation*locus interaction factor that has the opposite effect. The new weights of *x*_2_ and *x*_6_ found by rerunning the optimisation routine confirm the predictions given in (3) and (4). Note that the parameters estimating the intrinsic processing speed at each position (as obtained in (1)) are *not* altered by the weight reassignment, precisely because the changes of weight in the elevation factor and elevation*locus factor are self-cancelling.

Turning to the models of each individual subject and applying the equivalent weight re-assignment to the elevation*locus interaction factor, the main effect of elevation factor is now found to act consistently across subjects (i.e. acting to favour the inferior target positions in all subjects). Hence, the main outcome of this first weight reassignment within the model is that all factors and interaction terms now act consistently across subjects (with the associated chance probability decreasing to 2.9 × 10^−11^).

#### 3.4.3 Model parameters: (b) item density

The basic supposition regarding the effect of display mode upon search speed, at each position, is that search is carried out independently within each hemisphere, amongst the items that a single hemisphere is capable of seeing; the number of items would be smaller in bilateral displays (depending upon the proportion of items that have an ipsilateral representation). This variable, which can be labelled item ‘density’, should be captured by the product of the main effect of display, and the display*elevation interaction; inclusion of the latter interaction factor recognises that the ipsilateral representation in superior and inferior hemifields may be dissimilar. For both factors, the effect upon unilateral modes is modelled as 1.00. As mentioned above, the main effect of display favoured bilateral mode and the interaction was also consistent across subjects, taking the form of a faster search speed for inferior bilateral displays, and a slower search speed for superior bilateral displays. The product of the relevant modes of these factors produces a pair of ‘density’ factors, *D*_supr_ and *D*_infr_, specifying the reduced item density contingent upon bilateral display mode.

(5) $$x_{1}=B\;\text{and}\;U=1$$

(6) $$x_{4}=BI\;\text{and}\;x_{4}^{-1}=BS\;\text{and}\;US=UI=1$$

(7) $$D_{\text{supr}}=B\times BS=x_{1}\times x_{4}^{-1}\;\text{and}\;D_{\text{infr}}=B\times BI=x_{1}\times x_{4}$$

#### 3.4.4 Model parameters: (c) crowding factors

The remaining factors to be incorporated in the model are the display*locus, and display*elevation*locus interactions. Both factors include locus, and are not interpretable as global effects of item density, in that the ipsilateral representation of distractors in bilateral displays cannot be affected by switches in target position from V to H locus within a quadrant. There may, however, be local density affects akin to crowding. This is because, in all subjects, the target positions disfavoured by the locus*display interaction were the H locus in unilateral displays, and the V locus in bilateral displays. These loci are relatively central in their respective displays, with a shorter average distance to all other items (in 4- and 8-item displays, that is).

The effect of the display*locus, and display*elevation*locus factors upon the non-crowded loci (unilateral V and bilateral H) was set to 1.00, in both the 2-way and 3-way interactions, allowing the crowding effect to be modelled as a specific effect upon search speed at the crowded loci (bilateral V and unilateral H). Thus, the 2-way interaction specified a uniform speed reduction for the bilateral V-loci and unilateral H-loci (8), that the 3-way factor enhanced at both inferior loci, and counteracted at both superior loci (9). Or, in other words, the model specified two crowding factors *C*_supr_ and *C*_infr_ (10) with the optimisation routine typically returning *C*_infr_ > *C*_supr_ > 1.00 in subject and group data.

(8) $$x_{5}=BV=UH\;\text{and}\;BH=UV=1$$

(9) $$x_{7}=BIV=UIH\;\text{and}\;x_{7}^{-1}=BSV=USH\;\text{and}\;BIH=UIV=BSH=USV=1$$

(10) $$C_{\text{supr}}=UH\times USH=x_{5}\times x_{7}^{-1}\;\text{and}\;C_{\text{infr}}=UH=UIH=x_{5}\times x_{7}$$

#### 3.4.5 The development of a ‘speed’ model

To summarise the above, performance at each target position is specified by the following:

$$\text{RT slope}=S_{EL}\times D_{E}\times C_{E}$$

where *S*_*EL*_ is the intrinsic processing speed for target positioned at specified elevation and locus; *D*_*E*_ is the density coefficient at specified elevation, for bilateral displays (else = 1); *C*_*E*_ is the crowding coefficient at specified elevation, for V-locus in bilateral displays and H-locus in unilateral displays (else = 1).

The values obtained for intrinsic speed, density and crowding parameters for the group, and individual subjects, are reported in Table 1. The speed values lie within the range of RT slopes observed for unilateral and bilateral displays at each position, as would be expected. With one exception (*C*_supr_ = 0.83 in subject #4) the crowding values are above 1.0, confirming the ‘crowding’ interpretation of a relative slowing of search speed at the relevant loci in 6/6 cases in one quadrant, and 5/6 in the other. The central aim of the exercise, however, is to gauge the effect of display mode in terms of the density parameter: *D* < 1 is consistent with the inference of a bilateral advantage in search speed. The outcome shows that this applies to all six subjects with *D*_infr_ < 1, and three subjects with *D*_supr_ < 1; the three subjects where *D*_supr_ > 1 prove to be exceptions. Nominally, the parameter *D* signifies the relative number of items seen by the target hemisphere, in bilateral vs. unilateral displays, that may vary with the strength of an ipsilateral representation. From neurobiological considerations, it is unlikely that the ipsilateral representation could ever outweigh the contralateral representation, as *D* > 1 would imply, and in this respect the model evidently requires adjustment.

The modification is prompted by the notable observation that, in all subjects, *D*_supr_ > *D*_infr_, which implies that the ipsilateral representation of superior field outweighs that of inferior field. This raises a quandary for, although the target is likely to be represented in both hemispheres, the model has so far assumed that the target is always found by the search process in the contralateral hemisphere. But if the ipsilateral representation is characterised by a greater weight for superior quadrant items than inferior quadrant items, the target could enjoy a greater competitive advantage in the ipsilateral hemisphere – in the particular case of a superior target in unilateral presentations. Note that, as shown by Fig. 3, there is a speed advantage, on average, for superior compared to inferior target locations in unilateral displays. To accommodate this ‘ipsi-hemisphere’ solution in the model, it is necessary to re-assign some of the weight of the display*elevation factor to its *US* mode. Modifying Eq. (6):

(11) $$x_{4}=US\;\text{and}\;x_{4}^{-1}=BS\;\text{and}\;BI=UI=1$$

The weight of the display*elevation factor is now applied equally between BS and US, with opposite effect on each, whilst the definitions of *D*_supr_ and *D*_infr_ (Eq. (7)) are unchanged. Appendix B describes this modification in more detail.

The outcome of the revised formulation (‘Model 2′) is shown in Table 2. It reduces *D*_supr_, such that *D*_supr_ < 1 in all subjects, abolishing the unlikely inference that the ipsilateral representation might outweigh the contralateral representation. There are compensating decreases in the intrinsic processing speed parameters for the two superior loci (NB. – Table 2 shows *increased* numerical values, because it reports time per item); essentially, the revised formulation of the model is consistent with ‘fewer’ items to be processed in dealing with superior unilateral targets and, as the observed search speed is fixed, the underlying processing speed must decrease. The other five parameters are unchanged.

#### 3.4.6 Specifying interhemispheric transfer

The D parameter signifies a lower item density in the representation of a bilateral display within the hemisphere contralateral to the target, contingent upon a partial representation of ipsilateral space or, perhaps, a weaker weight of representation for the ipsilateral half of the display. Either way, it is a simple step to replace *D* with another parameter, *T*, that directly specifies the effective, proportional reduction in weight of the ipsilateral representation, as compared to the contralateral representation: *D* = (*T* + 1)/2 and hence:

(12) $$T_{\text{supr}}=2D_{\text{supr}}-1\;\text{and}\;T_{\text{infr}}=2D_{\text{infr}}-1$$

Because the ipsilateral representation is thought to arise from callosal connections, *T* can be thought of as an interhemispheric ‘transfer’ parameter, whose value should lie in the range 0–1. The conversion of *D* to *T* values in Table 3 shows that this expectation is largely met, although there are two subjects (#4 and #5) where *T*_infr_ < 0. This anomaly rectifies itself with additional fine tuning of the model, as explained in Section 3.4.6.1.

The replacement of one parameter by another (i.e. replacing *D* by *T*) does not alter the model per se. But the explicit specification of transfer factors does provide further scope for modification, in at least two respects. Firstly (i), the allocation of the weight of the display*elevation factor in regard to the ‘ipsi-hemisphere’ solution can be specified in a more principled fashion; secondly (ii), the relative level of crowding in different display conditions can be adjusted to take account of the fact that weaker weights of ipsilaterally represented items should be less effective at inducing crowding.

##### 3.4.6.1 Redefining the weight of US mode in display*elevation factor

If, in the contralateral representation of a unilateral display, all items are allotted an equal weight, the ratio of total item weight to target weight is simply *n*, the number of display items. In the ipsilateral representation of the same unilateral display, the target has a weight *T*, or *T*_supr_ in the specific case of a superior target. The total item weight is 0.5*n* × (*T*_supr_ + *T*_infr_). Hence the ratio of total item weight to target weight is *n* × (*T*_supr_ + *T*_infr_)/2*T*_supr_. Thus the relative advantage for target search speed in the ipsilateral representation, when the target is superior, is given by the expression (*T*_supr_ + *T*_infr_)/2*T*_supr_. This leads to the following reformulation of factor mode weights (i.e. modifying (6)):

(13) $$x_{4}^{-1}=BS;\;\frac{\left(T_{\text{supr}}+T_{\text{infr}}\right)}{2T_{\text{supr}}}=US;\;BI=UI=1$$

where *T*_supr_ and *T*_infr_ are ultimately functions of *x*_*1*_ and *x*_*4*_, as derived from Eqs. (6), (7) and (12).

##### 3.4.6.2 The modification of crowding parameters

It can be assumed that all display items represented in a hemisphere are crowded to some degree; the crowding parameter *C* captures the enhanced level of crowding experienced by particular loci in each display format. So far, it has also been assumed that the crowding of the V locus in bilateral displays is no less than that experienced by the H locus in unilateral displays – but this will not be the case if half the items in bilateral displays have weaker weights on account of being represented by sub-maximal interhemispheric transfer. A suitable modification is to express crowding in bilateral displays as *C*^T^: the power function gives full crowding if *T* = 1, and no relative crowding if *T* = 0.

It is also pertinent to consider how crowding should be modelled in the ‘ipsi hemisphere solution’ for unilateral displays (i.e. in the specific case of superior targets). If *T*_infr_ = *T*_supr_ = 1, there would be no difference. But if *T*_infr_ < *T*_supr_ < 1, as inferred for all subjects, targets in the superior quadrant should experience less crowding by the more weakly weighted items in the inferior quadrant – although, not being fully weighted themselves, they might be more susceptible to crowding. An appropriate correction is therefore to use *C*^*T*_infr_/*T*_supr_^. The necessary modifications to the factor weights in the model are set out in Appendix C, leading to the final set of modelling equations given by Appendix D.

#### 3.4.7 Final model, and summary

Table 4 (‘Model 3′) summarises the joint outcome of the final modifications detailed in 3.4.6.1 and 3.4.6.2. Note the increased estimates of *T*_infr_ relative to Table 3, such that *T*_infr_ > 1 for all subjects. In fact, in comparison to the previous models’ outcome shown in Tables 1 and 2, all parameters bar one are altered (the exception being the intrinsic speed for the inferior V-locus, that equates directly to the observed search speed in the UIV condition, because this locus escapes both transfer and crowding effects).

**Fig. 5:**
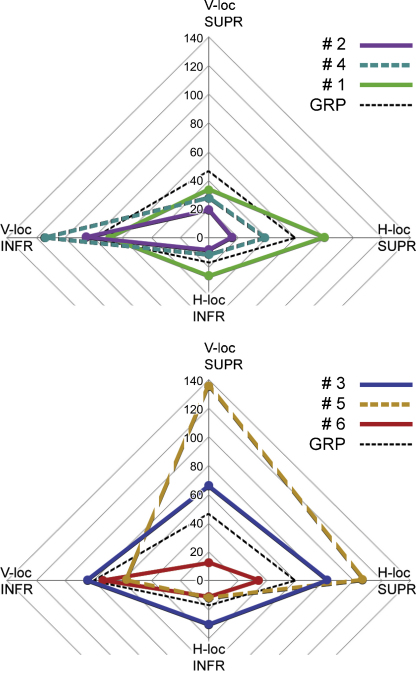
The modelled estimates of intrinsic processing speed (ms/item) at each target position for each subject, and the group outcome. Subjects are arbitrarily split into two sub-groups for display purposes. Note that the charts show inverse speeds (inspection time per item) so faster speeds are plotted more centrally. As shown by the group the effects of the factors elevation and locus are, in increasing order of magnitude (i) faster search speeds for targets at both H-loci; (ii) faster search speeds for targets at both inferior positions, and (as a factor interaction); (iii) faster search speed for a target at the superior V-locus and slower speed for a target at the inferior V-locus. These factors act the same way in all subjects, but their relative magnitudes can differ. For instance, in subject 5, the elevation factor is the most powerful.

The final set of estimates for the intrinsic processing speed at each target position is illustrated in Fig. 5. These values are analogous (but nonidentical) to the set of RT slopes obtainable for each subject from regression plots, if collapsing across the factors display, laterality and hand. A 2-way ANOVA (with factors of locus and elevation) applied to the model output shown in Fig. 5 gives a similar result to the raw data (Section 3.2): a significant main effect of locus (*F*(1,5) = 13.7, *p* = 0.014) and an elevation*locus interaction (*F*(1,5) = 51.6, *p* = 0.00081). Note that the factor elevation is not significant, despite having been modelled as a consistent effect favouring inferior target positions; it is simply masked, within ANOVA, by the interaction term acting in the opposing direction (as indicated in 3.4.2.1).

**Fig. 6:**
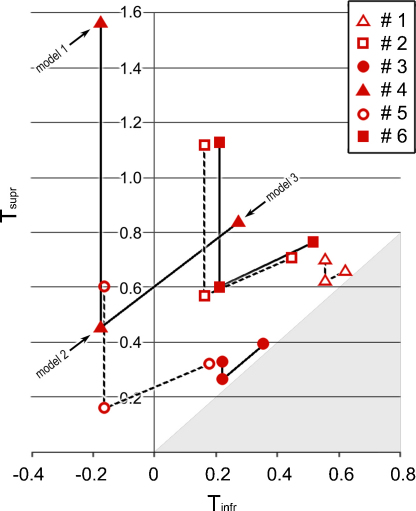
The development of transfer factor estimates (*T*_supr_ and *T*_infr_) in all subjects across models 1–3. Values of *T*_infr_ and *T*_supr_ for models 1 and 2 are converted from the tabulated values of *D*_infr_ and *D*_supr_ in Tables 1 and 2. Models 1, 2 and 3 are indicated for subject #4; the other subjects follow a similar pattern. All points lie outside the shaded region, denoting *T*_infr_ > *T*_supr_.

Fig. 6 shows how successive refinements of the model influence the estimates of the transfer factors *T*_infr_ and *T*_supr_ (after converting from *D* to *T* for the prior two model set-ups). In the third model, 0 < *T*_infr_ < *T*_supr_ < 1, for all subjects. The fact that 0 < *T* < 1 allows a viable interpretation of *T* as an interhemispheric transfer factor. And the fact *T*_infr_ < *T*_supr_ means that the motivation for the ‘ipsilateral hemisphere solution’ for target finding in the case of superior targets in unilateral displays is plausible across all subjects, and not just the trio in whom *T*_supr_ > 1 when the standard ‘contralateral hemisphere solution’ was applied.

To summarise the modelling exercise, the model replicates the RT slopes of the search-efficiency functions shown in Fig. 3, but as individual subject as well as group data. These eight functions specify the effect of three binary factors – target locus, target elevation, and unilateral vs. bilateral display mode. The laterality factors (right or left hand responses, and target locations) were not found to be significant, and are collapsed. An 8-fold set of data allows, maximally, a model with 8 parameters – ultimately, four locus-specific processing speeds, two ‘crowding’ factors, and two ‘transfer’ factors. Or, starting with the statistical factors, a single base speed plus three main-effect factors, three 2-way interactions and one 3-way interaction. Sections 3.4.1–3.4.6 elaborate how one set of factors derives from the other. The factors do not have to be multiplicative, as the data can be modelled using additive effects, or logarithms, or anything else. However, a multiplicative factorial model is consistent with the use of log-transformed data in the ANOVA. Essentially, the raw statistical factors describe the influence of display geometry and the final model factors attempt to isolate elements of neural processing (i.e. intrinsic processing speed, neighbourhood crowding and interhemispheric transfer).

### 3.5 Target-absent trials

Target-absent trials were characterised by just two factors, display and hand. Clearly, there is no target locus factor, and the display characteristics elevation and laterality are also analysed separately (as unilateral displays are no longer sub-categorised by ‘elevation’, nor bilateral displays by ‘laterality’). The two remaining binary factors produce four different conditions and associated RT slopes, each derived from 72 RT observations per subject, after pooling data from left and right unilateral, or superior and inferior bilateral displays. The 2-way ANOVA applied to the target-absent data is therefore compiled from 24 estimates of RT slope across all subjects. The outcome reveals no effect attributable to display, or hand, or their interaction; in particular, for the main effect of display, *F*(1,5) = 0.8, *p* = 0.41.

As illustrated by Fig. 2, the group average ratio of RT slope between unilateral and bilateral target-absent displays is 1.06. Individual variation in performance was examined in similar plots. Across subjects, the UNL:BIL ratio of RT slope varied from 1.21 to 0.87, bilateral search speed actually being slower in two individuals. In no case was there a significant difference in RT slope, as assessed by ANCOVA on the trial-by-trial data; the two tail-end cases noted above gave, respectively, *F*(1,270) = 2.1, *p* = 0.16 and *F*(1,271) = 2.7, *p* = 0.099.

#### 3.5.1 Hemifield variability in target-absent trials

**Fig. 7:**
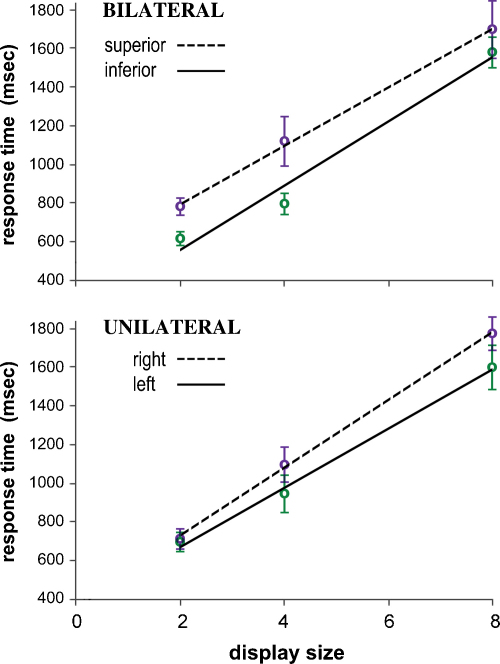
Response time plots for pooled subject data under the target-absent condition, showing the effects of (above) superior and inferior hemifield presentation in bilateral trials, and (below) right and left hemifield presentation in unilateral trials. Error bars indicate ± s.e. of subject means.

The subsidiary effects of elevation and laterality within bilateral and unilateral display modes are shown in Fig. 7. Separate ANOVAs (with factors of elevation and hand for bilateral trials, and laterality and hand for unilateral trials) again failed to reveal a firm influence upon RT slope by either display factor; at most, there was a possible trend toward faster item scanning in left sided unilateral trials [*F*(1,5) = 4.6, *p* = 0.084]. There was no interaction between hand and laterality in the unilateral condition [*F*(1,5) = 0.1, *p* = 0.77]. The notable gap in the response times expressed by each pair of functions, attributable to a difference in the fixed component of RT, will be examined in 3.6.

The hemifield location of target-absent displays provoked more significant individual variation in performance than the quality of being unilateral or bilateral. The ratio of RT slope for left v. right unilateral displays varied from 0.71 to 1.12, with three subjects significantly faster on the left, as assessed by ANCOVA [*F*(1,135) = 4.9, *p* = 0.028; *F*(1,132) = 7.9, *p* = 0.0057; *F*(1,133) = 4.9, *p* = 0.028]. The ratio of RT slope for superior vs. inferior bilateral displays varied between 0.50 and 1.08, with two subjects significantly faster superiorly [*F*(1,135) = 11.8, *p* = 0.00077; *F*(1,130) = 5.6, *p* = 0.020].

### 3.6 Analysis of simple response time

Previous studies of target location in search paradigms (and all previous demonstrations of a bilateral field advantage) have worked mainly in terms of simple response time rather than an item scanning speed (Efron & Yund, 1996; Previc & Blume, 1993). The equivalent variable from the present study is the mean response time (mRT), i.e. the interpolated RT for 4.67 distractors (the ordinate value at which ANCOVA assesses the vertical displacement of two regression lines). Group results are briefly presented in this format to afford comparison with previous work, concentrating on possible quadrantic field biases.

Variations in mean RT with display conditions were analysed using ANOVAs equivalent to those applied to RT slope. The 5-way ANOVA for target-present conditions mirrored the chief effects upon scanning speed. Firstly, a faster response to bilateral displays, 622 vs. 660 ms [*F*(1,5) = 33.6, *p* = 0.0022]. Secondly, effects of locus [*F*(1,5) = 16.2, *p* = 0.010] and locus*elevation [*F*(1,5) = 43.8, *p* = 0.0012], producing RT variation across target position (averaging across display mode) as follows: inferior H-locus (580 ms), superior V-locus (622 ms), superior H-locus (650 ms), inferior V-locus (712 ms). Unlike the analysis of RT slope there was a mild effect linked to faster responses by the right hand, 627 ms vs. 655 ms [*F*(1,5) = 6.8, *p* = 0.048], but once again there was no interaction between hand and laterality [*F*(1,5) = 0.9, *p* = 0.38], nor any other higher order interactions.

The analysis of mRT under target-absent conditions revealed a slight, insignificant advantage for bilateral displays (1098 ms vs. 1138 ms) [*F*(1,5) = 1.3, *p* = 0.31], and no effect of superior or inferior presentations within bilateral mode. The latter inference prevailed [*F*(1,5) = 3.2, *p* = 0.13], despite the group average mRT for superior displays being markedly slower (1198 ms vs. 1000 ms – as evident in Fig. 7). Unilateral trials showed a mild advantage for left-sided displays, 1079 ms vs. 1198 ms [*F*(1,5) = 5.5, *p* = 0.065] and for right-hand responses: 1116 ms vs. 1161 ms [*F*(1,5) = 6.6, *p* = 0.050]. There was no hand*laterality interaction [*F*(1,5) < 0.5, *p* > 0.5), as might have signified a tendency toward faster responses by the contralateral hand.

Examination of individual performance in the bilateral, target-absent trials revealed substantial variation, in that three subjects had significantly faster responses to inferior displays, by 300–600 ms, but one showed the reverse (slower by 121 ms). Also four subjects significantly favoured the left-sided versions of unilateral displays, and none the opposite. The following analysis was conducted in order to probe the origin of these individual variations more closely, by examining their co-occurrence under target-absent and target-present conditions.

### 3.7 Cross subject correlation of hemifield bias under target-absent and target-present conditions

To compare hemifield bias (in both the horizontal and vertical axes) the target-present trial data were collapsed across target locus to provide a single measure of performance matching the target-absent conditions; thus, for example, a single RT plot for superior hemifield search was derived from data collected for targets at V- and H-loci in both superior quadrants. Data was further collapsed across the response factor hand, given non-significant hand*elevation and hand*laterality interactions in ANOVA of both target-present and target-absent data. The parameters RT slope and mRT were then extracted to characterise scanning speed and mean response time in each hemifield in each subject, with and without the presence of a target.

**Fig. 8:**
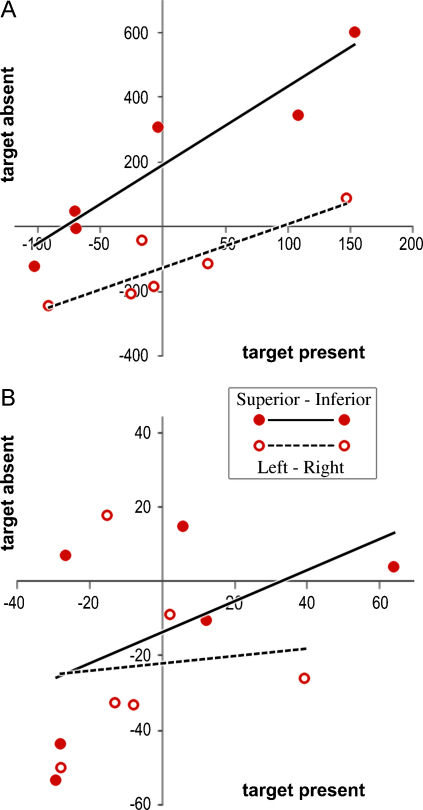
Correlation across subjects of hemifield asymmetries in search performance with and without a target. Search performance is characterised by the mean response time in A (ms), and by RT slope (i.e. item scanning speed) in B (ms/item). Asymmetry in search performance is represented by the difference in these parameters, i.e. superior–inferior hemifield and left–right hemifield, as respectively observed in bilateral and unilateral display modes. Each plot shows the correlation between performance under target-absent and target-present conditions. Only the correlations shown in A (for mean response time) are significant: left–right, *R*^2^ = 0.77, *t* = 3.7, *p* = 0.02; superior–inferior, *R*^2^ = 0.90, *t* = 6.1, *p* = 0.004). The respective regression equations are; *y* = 1.4*x* − 128 and *y* = 2.4*x* + 191.

The results of this exercise are simply summarised. There was no significant correlation of search performance with and without a target – for either performance parameter in any of the four hemifields. Nonetheless, the correlation coefficients were all positive, and ranged from 0.44 to 0.67 (mRT) and 0.24 to 0.62 (RT slope). By contrast, there was a robust correlation for the *difference* in performance between hemifields, at least for the mean response time. This was true for both superior vs. inferior and left vs. right, as shown in Fig. 8a; scanning speed behaved differently, showing negligible correlation (Fig. 8b). Comparison of the differential performance across hemifields thus enhanced the correlation coefficient for mRT (*R*_LEFT–RIGHT_ = 0.88 and *R*_SUPR–INFR_ = 0.95) but not for RT slope (0.10 and 0.54). The nature of this outcome implies that the mechanism(s) contributing to hemifield asymmetry may vary in their direction of action, across subjects, and also affect a fixed component of response time, as opposed to modulating the item scanning speed.

## 4 Discussion

### 4.1 Overview

Each of six subjects tested with a difficult visual search task showed a significant bilateral advantage for target-present, but not target-absent conditions – a result that may be rationalised by consideration of the relative operations of inter- and intra-hemispheric circuitry within the context of a guided search strategy (Cave & Wolfe, 1990; Chun & Wolfe, 1996; Muller-Plath & Pollmann, 2003; Wolfe, 1994) that couples together parallel and serial processes.

The paradigm was adapted from a previous study of split-brain subjects, that had demonstrated an approximate twofold increase in search speed for bilateral vs. unilateral arrays of display items (Luck et al., 1989, 1994). The modified displays were geometrically regular, with search items occupying a single hemifield (superior or inferior for bilateral displays, right or left for unilateral) and targets placed at one of two fixed positions per quadrant. The outcome revealed notable asymmetries in performance in superior or inferior field, coupled to still stronger effects related to target position within a quadrant. In order to isolate the bilateral vs. unilateral component of performance from other aspects of display geometry, and target position, it was necessary to develop a numerical model for the interaction of these factors in governing search speed, measured as the slope of the RT function (or equivalently, the inspection time per item (ITI)). Bilateral advantage in target detection was then parameterised by an estimate of the reduction of the number of display items ‘seen’ by a single hemisphere under bilateral display conditions.

If this seems to imply a serial mechanism, the observation that search performance lacks a bilateral advantage in the absence of a target immediately poses something of a conundrum. Clearly, whatever the neural apparatus implementing the model, the number of items that it ‘sees’ should not depend on whether one of these items happens to be a target, or not. The two stages of a guided search system, however, might have sufficiently different operating characteristics as to be capable of the observed behaviour. The numerical model would take the part of the first parallel stage, weighting items to match their similarity to the desired target; the second, serial, stage inspects items in rank order of weight. Target search is relatively efficient, but in the absence of a target the weight distribution is more uniform, producing an outcome closer to random search, and a considerably steeper RT function. To suit the present data, the assumption would be that the first parallel stage is hemisphere based, and that the second is better (or perhaps fully) integrated across hemispheres. Thus a bilateral advantage will obtain if the weight of the target is enhanced when there are (effectively) fewer competing distractors within the parallel stage. In the absence of a target search is random and either exhaustive, or subject to a termination rule that is indifferent to the geometry of the stimulus array (Chun & Wolfe, 1996; Cousineau & Shiffrin, 2005).

From this perspective, it would be logical to adapt the numerical model derived above so that it prescribes how stimulus geometry (acting through interhemispheric transfer, and crowding factors) modulates the weight of an item at a particular locus, with an added formalism for translating weight into search speed. The existing model is sufficient for present purposes, of distinguishing bilateral advantage from other aspects of display geometry, and discussion here aims to consolidate the outline given above, relating the present work to previous psychophysical insights regarding interhemispheric integration.

### 4.2 Effect of target position

Target position was, unexpectedly, the dominant factor governing the apparent processing time per item (i.e. RT slope). In a parallel model, this would point to position-dependent competitive bias (Efron & Yund, 1996). The group data revealed no consistent distinction between right and left positions, leading to the following ranking of four generic target locations, in order of decreasing efficiency: (1) inferior H-locus; (2) superior V-locus; (3) superior H-locus; (4) inferior V-locus. From the performance data, it is arguable to invoke at least three effects to account for this rank ordering of search speed across positions: *(a)* a locus effect favouring the H-loci over the V-loci; *(b)* an elevation effect favouring inferior field positions over superior field positions; *(c)* most powerfully, a locus*elevation interaction effect favouring the superior over the inferior V-locus whilst not affecting the H-loci.

Target eccentricity is a known factor influencing response time in search tasks (Carrasco, Evert, Chang, & Katz, 1995; Previc & Blume, 1993). This reflects a tendency to deploy attention to more central items before more peripheral ones or, in other words, to search from central field outward (Wolfe, O’Neill, & Bennett, 1998; Woodman & Luck, 2003). There is also a potential contribution from lower level factors, such as visual acuity, since the effect of eccentricity under some conditions can be moderated by scaling stimulus size in accord with cortical magnification (Carrasco & Frieder, 1997; Carrasco & Yeshurun, 1998). The targets in the present experiments were all of equal eccentricity, but it is possible that the position effects were related to meridional variations in the eccentricity dependency of these cognitive or visual factors.

#### 4.2.1 Visual factors

A number of previous studies have suggested a search priority for items located in the superior and/or right hemifields (Previc, 1996; Previc & Blume, 1993; Yund et al., 1990a; Yund, Efron, & Nichols, 1990b). Where the target was a grating of specified orientation, the right-field advantage was thought to reflect higher acuity within this hemifield (Yund et al., 1990a, 1990b). A subsequent study by this group showed that a simple bar target showed less of a field effect, and more of a position effect – being located faster near to the vertical meridian when vertically oriented, and near the horizontal meridian when horizontally oriented (Efron & Yund, 1996). The key feature now appeared to be a radial (or near radial) alignment of the target orientation. When a coloured target was used, the position and field effects were abolished. These studies imply that at least some position effects in search depend crucially upon the nature of the target/distractor discrimination.

In the present study the coloured components of the display items shared a horizontal contour, so it is possible that an analogous radial field alignment factor was in play. And, since the aim of the search was the detection of ‘blue-on-topness’, it is worth considering what is known of variations in SW (shortwave) sensitivity, which include better sensitivity in lower field for SW perimetry (i.e. flash detection) and meridional variations in SW acuity (Beirne, Zlatkova, & Anderson, 2005; Demirel & Robinson, 2003; Sample, Irak, Martinez, & Yamagishi, 1997). The latter include higher acuity at horizontal over vertical meridian locations, and for inferior over superior vertical. Superficially, at least, these regional variations mirror both effects *(a)* and *(b)* above. For cautionary purposes, it should also be noted that the SW perimetry and acuity measurements were made at eccentricities greater than the target locations in the search task (minimally, at 10° in the study of Beirne et al. (2005)) and revealed a trend of decreasing regional variation toward central field.

#### 4.2.2 Cognitive factors

One way to rationalise the priority accorded to central target locations, in the context of a guided search model, is that the greater magnification of central stimuli within cortical maps endows those central locations with a competitive advantage over more peripheral locations (Wolfe et al., 1998). A similar kind of logic would then infer that the lesser attentional resolution in superior field (He et al., 1996; Intriligator & Cavanagh, 2001) is also indicative of a quadrantic disadvantage for superior targets competing with inferiorly located distractors, with a consequent lessening of search speed for superior field target locations – consistent with effect (b), above.

The more taxing problem is to account for the opposite effect, (c), producing an imbalance at the twin V-loci, but now favouring the superior position. The bilateral advantage paradigm of Awh and Pashler (2000) produced strong asymmetry across the HM that might represent something similar. Specifically, subjects cued to report a pair of unilateral items presented simultaneously (under crowded conditions) at matching positions above and below the HM were much more accurate (e.g. 88% vs. 51%) at the superior position. The items were located on the 45 diagonal, and hence intermediate between the present V- and H-loci. Nonetheless, this finding could be indicative of competitive advantage of superior over inferior locations that is more pronounced away from the HM, the basis of which is quite unknown.

### 4.3 Position effects and crowding

The effect of target position also interacted with display geometry, i.e. unilateral or bilateral. This effect took the form of a search-speed advantage for targets located on the fringes of the display – the V-locus in unilateral displays and the H-locus in bilateral displays. Although the model envisages a universal competition amongst all display items presented, this display*locus interaction can be interpreted as an indication that competition is fiercer between proximal display items than between distant items. Hence the model invokes a ‘crowding’ factor, slowing search speed at the positions with higher average proximity (the unilateral H-locus and the bilateral V-locus). Crowding so defined, showed, in turn, a mild interaction with elevation. The crowding effect was weaker on average in the superior field. Is this observation in conflict with the known, lesser attentional resolution of superior field (He et al., 1996; Intriligator & Cavanagh, 2001)? Not necessarily, because crowding here is modelled as the differential slowing of the more affected target locations; if superior locations have larger attentive fields (Intriligator & Cavanagh, 2001) and therefore experience greater crowding in general, this would not be reflected as an interaction with locus, but as a main effect of elevation.

### 4.4 The computation of bilateral advantage

The model, initially, represents bilateral advantage by a density parameter (*D*) signifying the reduced ‘number’ of items represented by a hemisphere under bilateral display conditions: in the hemisphere contralateral to the target a majority of distractors will be represented with reduced weight, attenuated by the pathways responsible for an ipsilateral representation, and hence their effective number is reduced.

The bilateral advantage in search speed was found to be greater within inferior field (or, in model terms, *D*_infr_ < *D*_supr_). The implied property of inferior field processing, that it is characterised by less interhemispheric integration, could be consistent with previous demonstrations of a bilateral advantage in respect of tracking tasks. The basic result is that bilateral presentation enables simultaneous tracking of twice as many items (Alvarez & Cavanagh, 2005). However, a follow-up study implied that there is greater interference (i.e. a slower threshold tracking speed, or less bilateral advantage) when twin tracked-items are separated across the superior VM (vertical meridian) than the inferior VM (Carlson et al., 2007).

The immediate inference would seem to be that interhemispheric connections providing the ipsilateral representation are correspondingly less dense, and/or less expansive, for inferior field. Somewhat surprisingly, the literature on callosal anatomy and physiology appears to lack conclusive evidence, either for or against this proposition. A superior/inferior asymmetry is not apparent in an fMRI study of ipsilateral field representation in human visual cortex (Tootell et al., 1998), for instance, but it is not clear if the comparative sensitivity of the imaging methodology is well matched to the present psychophysics. However, if the absence of any superior/inferior field asymmetry in callosal connectivity were an established fact, an alternative interpretation of the search speed asymmetry observed here would be to posit that the visual mechanism capable of detecting blue-on-topness might be instantiated at different levels in the serial pathways processing superior and inferior field content; i.e. that this mechanism is present at an earlier level in the inferior field pathway, at a less advanced stage of the serial expansion of ipsilateral representation, such that there is less competition from ipsilaterally represented distractors.

### 4.5 Fine tuning the numerical model

In its most basic form, the model has a linear multiplicative format, using eight parameters to exactly simulate search speeds at superior and inferior V- and H-loci under bilateral and unilateral display conditions (i.e. disregarding left vs. right hemifield differences, that were not significant). These parameters are an intrinsic processing speed for each of the four target positions, plus a pair of separate ‘crowding’, and ‘density’ parameters for superior and inferior field.

These parameters are initially somewhat abstract concepts, and several steps were taken to solidify their potential physiological significance. Firstly, the item density parameter *D* was replaced by an interhemispheric transfer parameter (*T*), on the basis that the reduction in *D* caused by bilateral displays is due to the lesser weight attached to ipsilateral (interhemispherically transferred) items: hence *D* = (*T* + 1)/2, with the expectation that 0 < *T* < 1.

The introduction of *T* facilitated several additional modifications. One of these was to allow superior targets in unilateral displays to be ‘found’ by the ipsilateral hemisphere (see 4.5.1) – a consequence of the greater interhemispheric transfer in superior field than inferior field, meaning that superior targets would a enjoy a greater competitive advantage over inferior distractors in their ipsilateral representation. Another modification was to allow for the fact that crowding in bilateral displays should take account of interhemispheric transfer and would, for example, be of lesser magnitude in inferior field processing. Similar considerations also apply to the ipsilateral solution for unilateral superior targets, just mentioned. The crowding parameters were moderated by applying *T* as an exponent (i.e. *C*^T^), introducing non-linearities to the model.

The final outcome of the modelling process provided a reasonable parameterisation for each individual subject, as well as the group data. The latter gave *T*_infr_ = 0.42, *T*_supr_ = 0.53, *C*_infr_ = 1.86, *C*_supr_ = 1.21; uniformly, across subjects, 0 < *T*_infr_ < *T*_supr_ < 1, and *C*_infr_ > *C*_supr_ > 1 (except one case where *C*_infr_ > 1 > *C*_supr_). In order to have any plausible physiological significance transfer factors are obliged to lie within the range of 0 to 1 and crowding factors should exceed 1, as was true in 23/24 parameters evaluated. The precise numerical outcome may be of questionable significance, and better evaluated on an ordinal than an interval scale; in other words, the comparative values should produce a meaningful ranking of subjects’ processing speeds, transfer and crowding characteristics. Furthermore, the fact that the asymmetry in the transfer parameter between superior and inferior fields was common to all subjects helps to establish it as a viable means of characterising the bilateral field advantage.

#### 4.5.1 Supporting evidence for the ‘ipsilateral hemisphere solution’

In terms of model mechanics the ipsilateral solution was implemented by allowing the weight of the display*elevation factor to distribute over the *unilateral superior* mode, as well as the bilateral modes. This had the general effect of reducing the estimate of *T*_supr_. It was particularly meaningful in the case of three subjects whose search speeds for superior targets showed a bilateral *dis*advantage. In a contralateral solution, this necessitated *T*_supr_ > 1, but the ipsilateral solution allowed *T*_supr_ < 1 in these subjects (as a value of *T*_supr_ > *T*_infr_ now models a relative acceleration of the superior unilateral search speed, as well as a relative deceleration of the superior bilateral speed). Studies of bilateral advantage conducted with fMRI show that unilateral stimuli do activate the ipsilateral hemisphere (Santhouse et al., 2002), including ventral occipital visual cortex in a letter-name comparison task (Pollmann, Zaidel, & von Cramon, 2003). Thus the ipsilateral solution is plausible, although direct imaging evidence is lacking as fMRI studies of visual search per se have yet to employ a bilateral advantage paradigm. There was an outside chance that the hemisphere responsible for target detection might be deduced from differing RTs between right and left hand, as an analogue of the CUD effect (‘crossed-uncrossed difference’) known to obtain in simple reaction time studies (Bashore, 1981). If so, the ‘ipsilateral solution’ would register in the present design as a display*elevation*laterality*hand interaction. Such an effect was not observed, nor any form of hand*laterality interaction, consistent with previous studies suggesting that a CUD dependent on interhemispheric transfer delay can easily be confounded by other factors, e.g. attention, or spatial compatibility of the manual response (Braun, Daigneault, Dufresne, Miljours, & Collin, 1995; Braun, Larocque, & Achim, 2004).

### 4.6 Parallel and serial models

**Fig. 9:**
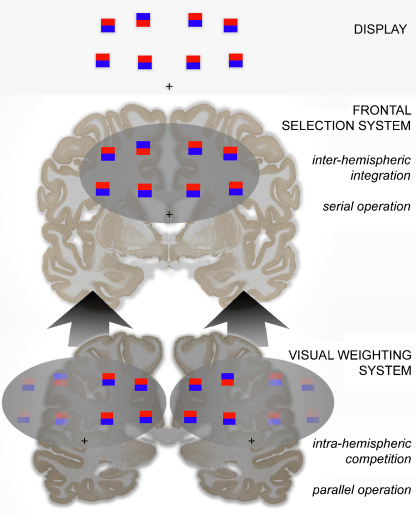
A ‘hopscotch’ implementation of guided search, alternating between single and dual hemisphere phases. In the first, parallel stage display items are weighted by mechanisms of intrahemispheric competition (e.g. through the interplay of occipital visual cortex and thalamus); the representations of ipsilateral items in bilateral displays are accorded less weight (signified by reduced contrast). The second stage of serial search is implemented by a bilaterally integrated ‘oculomotor’ network (e.g. frontal and parietal eye-field areas of cortex plus the superior colliculus, linked interhemispherically by crossed cortico-cortical, cortico-striatal, nigro-collicular and intercollicular connections). The anatomical separation of the two stages, as schematically depicted, should not mask the fact that the effect of attentional selection is itself mediated by re-entrant pathways to occipital visual cortex.

In the account given so far, the model parameterises bilateral advantage as a reduction in the number of items presented to one hemisphere, that then endows the target item (if present) with reduced levels of competition in comparison to a unilateral display. Clearly, this rests on two key premises: (i) that the mechanism underlying search is inherently parallel; (ii) that each hemisphere can perform this parallel operation in an autonomous fashion. Both can be retained if the model is held to represent not the whole search process, but the initial stage of a two-stage guided search process (Fig. 9). The output of the model should then be a weighted representation of each display item (rather than the ultimate search speed) that is used to guide a serial search process. In a revised model, this would entail replacing the intrinsic processing speed at each target position with an intrinsic weighting, that would be shaped by equivalent interactions with the *T* and *C* parameters. The advantages of a guided search model, as opposed to a purely parallel (or purely serial) system become more apparent when attempting to account for the lack of a bilateral advantage in the target-absent conditions.

A simple serial model, in which a stimulus array is scanned by a single focus of attention, provides a natural explanation for the outcome in split brain subjects, if each hemisphere gains independent control of its own focus (Luck et al., 1994); this study did not report a target-absent condition, but would predict a similar result – a doubling of search speed in the bilateral condition. If normal subjects, however, scan with a single focus under dual hemispheric control, the serial model predicts no bilateral advantage in target detection. To do so, it would require an ad hoc assumption that attention shifts are faster between hemifields than within a hemifield, as if the hemispheres play a game of *ping pong* with the single focus. The available evidence, in fact, suggests exactly the opposite: 55 and 38 ms respectively for inter- and intra-hemifield shifts (Ibos, Duhamel, & Ben Hamed, 2009). An alternative formulation is that each hemisphere retains independent control of a single focus, even in an intact brain, and that enhancement of search speed for bilateral displays reflects a smaller number of items to search (fewer than the total but more than half, due to the ipsilateral representation). Even without recourse to the split-attention issue (Jans et al., 2010), this simple serial account can also be rejected as it fails to explain the loss of a bilateral advantage when the target is absent.

In contrast to serial models, the archetypal parallel strategy, e.g. as advanced by Duncan and Humphreys (1989) and modelled computationally (Deco, Pollatos, & Zihl, 2002) is that the relative weights of search-item representations gradually evolve in accordance to the match between each item and the stored template of the desired target. Ultimately, the largest weight attracts the focus of attention. Related formulations, such as ‘TVA’ (Bundesen, Habekost, & Kyllingsbaek, 2005) invoke a two-stage parallel process, whereby weights gathered in one set of units determine attentional selection mediated through a second set of units. However formulated, any such parallel process would almost inevitably possess some degree of hemispheric autonomy, given the known anatomy (e.g. the restricted callosal connections between early visual areas, and the near-total absence of interhemispheric cortico-thalamic circuitry). A bilateral field advantage would be the natural consequence. The problem faced by search models with purely parallel mechanisms is how to address target-absent conditions: essentially, an external decision-process must be invoked, which opts to quit after a period of time has elapsed roughly commensurate with successful target-detection trials in similarly sized displays. If so, the simplest expectation is that shorter RTs obtained with bilateral displays would be extended to the target-absent condition too, inconsistent with the present observations.

The two-stage hybrid model copes more readily with the present observations, given the single proviso that the second serial stage is more bilaterally integrated than the first parallel stage. If a target is present, search efficiency benefits from less competition within the parallel stage under bilateral presentations, as outlined above. The serial stage – which is roughly equivalent to the oculomotor system, according to the premotor theory of attention (Awh et al., 2006; Corbetta, 1998; Moore et al., 2003; Rizzolatti et al., 1987) – receives multiple bids from the visual cortex of both hemispheres, and the correct target may, or may not be the first item selected, depending on the level of internal noise (Herd & O’Reilly, 2005; Wolfe, 1994). In the absence of a target any ‘guidance’ to target location reflects internal noise alone, and serial search becomes a random process. It might be exhaustive, or items might be inspected down to a certain threshold activation above background (Chun & Wolfe, 1996; Cousineau & Shiffrin, 2005). Either way, there is no expectation of a bilateral advantage in the target-absent condition.

For ease of reference – and in alternating from a single to a dual hemisphere process – this modification to guided search might be termed the ‘hopscotch’ variation. The schematic in Fig. 9 summarises the key points of its operation and nominates some likely neural circuitry.

### 4.7 Relation to previous demonstrations of the bilateral field advantage

Perhaps surprisingly, the present account of a bilateral advantage in visual search may be a distant relative to previous demonstrations in respect of item discrimination tasks. These previous studies have typically used twin cues to summon simultaneous attention to two locations, and this evidence for a bilateral advantage also feeds into the split attention debate, suggesting that dual foci of attention are easier to produce under independent control from each hemisphere (see 1.1). The use of cues ensures a top-down allocation of attention, prior to target stimulus onset; furthermore the positions used were typically invariant across a class of trials, so subjects can become relatively practised at allocating attention in a fashion that maximised performance. It has been suggested that twin foci of attention are more likely to be elicited by such task-specific conditions (Jans et al., 2010).

In the search task, by contrast, target positions were unpredictable prior to stimulus onset. There were 8 potential target locations (and subjectively up to 16, as subjects were not told that the extra 8 locations used in the 8-item trials were never target locations). Whilst the target was not salient enough to draw attention in classical bottom-up manner, the display would be expected to guide attentional deployment with a greater visual influence than in a cued dual-target paradigm. As outlined above, the results can be accounted for by a standard guided search model, controlling a single attentional focus. The absence of a bilateral advantage in the target-absent condition argues against the use of a split focus of attention in this task.

### 4.8 Relation to previous split brain inferences regarding interhemispheric integration of attention

The arguments above (4.6) rejected the simple serial search account for the observations of Luck et al. (1989), Luck et al. (1994) in split brain subjects. But the guided search model provides a workable substitute, in that the bilateral display puts the target into competition with fewer than half the distractors of a unilateral display, in the first parallel stage, consequently enhancing the relative weight of the target's bid for selection by the second, serial stage. Note that the split-brain subjects are still slower overall, even for the bilateral condition. This is to be expected, given that the serial search system has been damaged by the loss of its interhemispheric integration.

Afraz, Montaser-Kouhsari, Vaziri-Pashkam, and Moradi (2003) report a similar kind of experiment in a subject (MD) with a partial, posterior callosectomy. Unlike a control group, MD recorded similar response times to a conjunction search task when faced with a unilateral display, or a bilateral display with double the number of search items. As no further variation in set size was used, bilateral and unilateral scanning speeds were not measured, but the demonstration was sufficient to infer that MD's response was little affected by distractors in the hemifield opposite to the target. If MD's visual cortex was effectively ‘split’, the bilateral condition would not present the search target with additional competing distractors, and the target weighting would not have lessened. The frontal serial system may have been intact, or at least partially so. But even if acting as single unified system in receipt of six bids from the six display items in the bilateral condition, the weight of the target bid, and the probability of selection, could be much the same in both bilateral and unilateral conditions.

The same report describes another experiment in which, conversely, MD's performance was indistinguishable from the control group (Afraz et al., 2003). Here the target occupied a fixed location in superior field, 1° from the midline, and was either crowded, or not, by items continuing an arc of similar eccentricity into the opposite hemifield. MD's ability to identify the target showed a normal susceptibility to crowding, despite its origination from the opposite hemisphere. In contrast to the search task, this experiment seems to have demonstrated interference mediated by MD's intact frontal interhemispheric contacts. The difference, of course, is that the target location was not found by visual guidance, but by the subjects’ internal knowledge. Thus attentional selection was restricted to frontal operations, whose only deficit would be the loss of each lobe's representation of ipsilateral display items. With intact contralateral representations, the competitive balance between hemispheres in MD may not have been disturbed, in contrast to the search task, allowing task performance much as normal.

### 4.9 Sources of discrepancy with previous work

**Fig. 10:**
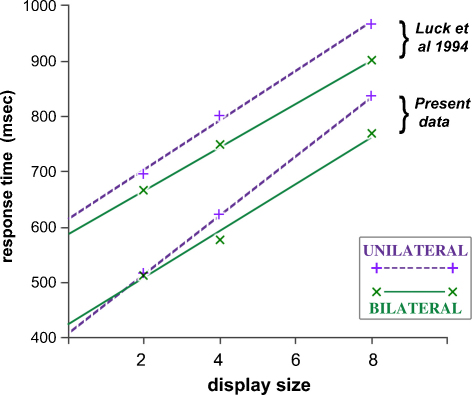
Comparison of the data from this study (as shown in Fig. 1, target-present) to the outcome for the control group (i.e. normal subject data) of a similar bilateral vs. unilateral search task from Luck et al. (1994).

Fig. 10 compares the current outcome to control data presented by Luck et al. (1989) and Luck et al. (1994).^1^ The ratio of unilateral:bilateral slope is not much different (1.25:1 here; 1.13:1 previously), but sufficient to alter the statistical assessment. It should be noted that a ratio of 1.13 is within the range of variation associated with different target positions observed in the present study, being typical of a superior hemifield location (see Fig. 3). Hence, although the conclusions may differ, the underlying observations are not so discordant. Note that the present participants also recorded faster average responses, by as much as 200 ms. Thus it is possible that levels of subject training, random vs. regular display geometry and variations in procedure may all have contributed subtly to the different outcomes (see 1.4 and 2.2).

### 4.10 Spatial bias in fixed or variable components of search time: intersubject variability

Guided search is efficient, in that typically a target is found with fewer than the 0.5*(*n* + 1) item inspections that characterise a random search (*n* = display size). An estimate of basic item scanning speed, the inspection time per item (ITI) can be taken from search speeds in the target-absent condition,^2^ where RT slopes were not much affected by different display conditions. Individual subjects typically showed a 10–20% variation in the ITI for left vs. right or superior vs. inferior hemifield, as measured in target-absent conditions. When a target is present, variations in search efficiency (i.e. variations in RT slope) across different target loci – and across subjects – are more substantial, and this could reflect the various factors determining internal item weightings, as discussed above (4.2). In other words the ITI might be relatively consistent, but targets with lesser weightings require a greater number of item inspections, on average, before they are found. From this perspective, it is not unexpected that search efficiency with and without a target fails to correlate across subjects (Section 3.7): the factor affecting both forms of search, ITI, is consistent and most variability is contributed by the factor affecting target search alone, item weighting.

The alternative measure of performance, mean response time, includes a number of ‘fixed’ components in addition to item scanning time. These include visual latency, decision time and motor delay. Decision time is one component that might be anticipated to be longer, and more variable, under target-absent conditions, depending on a subject's criteria for terminating search (Chun & Wolfe, 1996; Cousineau & Shiffrin, 2005). Hence it is no less unsurprising that mean response time with and without a target also fails to correlate across subjects, at least when measured within a specified hemifield.

Interestingly the two measures, efficiency and mean response time, behave quite differently when what is correlated (i.e. tested for correlation in the presence or absence of a target) is not performance within a given hemifield, but the performance difference between hemifields (Fig. 8). The field difference in mean response time (but not search efficiency) now shows a robust correlation across subjects, for both superior vs. inferior and left vs. right. This indicates that a subject's hemifield bias affects a fixed component of response time, operating under both target-absent and target-present conditions. Is this an early or a late component? An early event, such as an attentional ‘engagement time’ (the time taken to switch attention from fixation to the first item inspected) should not distinguish the two conditions, but in fact the relationship between the target-absent and target-present response times (specified by the regression equations for Fig. 8a) is not a simple equality. The hemifield bias therefore appears to reflect a variable decision time, dependent on the hemifield and the presence or absence of a target.

To recapitulate, the present study isolates two effects of target position: one relating to search efficiency (i.e. RT slope), which is sufficiently regular across subjects to be captured in the group data (e.g. Fig. 3); and a second, captured in the mean response time and inferred to relate to a decision time, that is quite variable from subject to subject (as shown in Fig. 8a). A number of previous reports have discussed various field asymmetries in search tasks, and reported *consistent* findings amongst groups of subjects (Previc, 1996; Previc & Blume, 1993; Yund et al., 1990a, 1990b). These may have been reported, in the main, as simple response times (with use of a fixed set size), but the evidence here suggests that these were not rigid spatial attentional biases, favouring one or other quadrant, but rather demonstrations of regional variation in target-item weighting characteristics (subject to a plethora of cognitive and visual factors noted in 4.2) that modulate search efficiency at different target locations, and thus indirectly governed the response times obtained when set size was invariant. In a nutshell, the proposal is that the spatial biases affecting search efficiency are dynamic (the preferred locations depend on the nature of the target/distractor discrimination) but are relatively consistent across subjects performing an identical task; by contrast, the spatial bias affecting decision making is more task invariant but less consistent across subjects.

## Figures and Tables

**Table 1:** Modelling parameters as evaluated by Model 1.

Model 1	Infr V ms/item	Infr H ms/item	Supr V ms/item	Supr H ms/item	*D*_infr_	*D*_supr_	*C*_infr_	*C*_supr_
GRP	81	20	42	55	0.59	0.83	1.55	1.13
#1	69	27	33	79	0.78	0.85	1.19	1.04
#2	85	10	16	13	0.58	1.06	1.78	1.16
#3	84	34	63	87	0.61	0.65	1.25	1.28
#4	114	18	19	28	0.41	1.28	2.13	0.83
#5	57	17	107	89	0.42	0.80	1.65	1.20
#6	74	14	11	29	0.61	1.06	2.02	1.09

Infr V, Infr H, Supr V and Supr H are the intrinsic processing speeds at V and H loci in inferior and superior quadrants. *D*_infr_ and *D*_supr_ are density coefficients in inferior and superior quadrants; *C*_infr_ and *C*_supr_ are crowding coefficients in inferior and superior quadrants.

**Table 2:** Modelling parameters as evaluated by Model 2.

Model 2	infr V ms/item	infr H ms/item	supr V ms/item	supr H ms/item	*D*_infr_	*D*_supr_	*C*_infr_	*C*_supr_
GRP	–	–	49	65	–	0.70	–	–
#1	–	–	34	82	–	0.81	–	–
#2	–	–	22	18	–	0.78	–	–
#3	–	–	66	91	–	0.63	–	–
#4	–	–	33	49	–	0.73	–	–
#5	–	–	148	123	–	0.58	–	–
#6	–	–	14	39	–	0.80	–	–

Blank entries are unchanged from Model 1 (as listed in Table 1).

**Table 3:** Conversion of density coefficients *D*_infr_ from Table 1 and *D*_supr_ from Table 2 to transfer coefficients *T*_infr_ and *T*_supr_ in the respective quadrants.

	*T*_infr_	*T*_supr_
*GRP*	0.19	0.40
*#1*	0.55	0.62
*#2*	0.16	0.57
*#3*	0.22	0.26
*#4*	−0.17	0.45
*#5*	−0.16	0.16
*#6*	0.21	0.60

**Table 4:** Modelling parameters as evaluated by Model 3. Conventions as for Table 1.

Model 3	infr V ms/item	infr H ms/item	supr V ms/item	supr H ms/item	*T*_infr_	*T*_supr_	*C*_infr_	*C*_supr_
GRP	81	17	47	60	0.42	0.53	1.86	1.21
#1	69	26	34	80	0.62	0.66	1.24	1.05
#2	85	8	20	16	0.45	0.71	2.21	1.25
#3	84	31	66	83	0.35	0.39	1.38	1.46
#4	114	12	28	39	0.27	0.84	3.28	0.73
#5	57	12	136	108	0.18	0.32	2.34	1.50
#6	74	11	13	35	0.52	0.77	2.53	1.13

## Appendix A

Observed RT slope

(A1) $$\text{USV}=x_{0}\times U\times S\times V\times US\times UV\times SV\times USV$$

(A2) $$\text{BSV}=x_{0}\times B\times S\times V\times BS\times BV\times SV\times BSV$$

(A3) $$\text{USH}=x_{0}\times U\times S\times H\times US\times UH\times SH\times USH$$

(A4) $$\text{BSH}=x_{0}\times B\times S\times H\times BS\times BH\times SH\times BSH$$

(A5) $$\text{UIH}=x_{0}\times U\times I\times H\times UI\times UH\times IH\times UIH$$

(A6) $$\text{BIH}=x_{0}\times B\times I\times H\times BI\times BH\times IH\times BIH$$

(A7) $$\text{UIV}=x_{0}\times U\times I\times V\times UI\times UV\times IV\times UIV$$

(A8) $$\text{BIV}=x_{0}\times B\times I\times V\times BI\times BV\times IV\times BIV$$

## Appendix B

In the initial weight allocation for the factors display and display*elevation:

(5′) $$x_{1}=B\;\text{and}\;U=1$$

(6′) $$x_{4}=BI\;\text{and}\;x_{4}^{-1}=BS\;\text{and}\;US=UI=1$$

(7′) $$D_{\text{supr}}=B\times BS=x_{1}\times x_{4}^{-1}\;\text{and}\;D_{\text{infr}}=B\times BI=x_{1}\times x_{4}$$

Hence, *D*_supr_ can be substituted into Eqs. (A2) and (A4), and *D*_infr_ into (A6) and (A8).

The optimisation routine obtains *x*_1_ < 1 and *x*_4_ < 1 in all subjects, characterising the main effect of display as an increased speed for the bilateral condition, and the interaction as a further acceleration for the inferior bilateral condition, and a deceleration for superior bilateral condition. It then follows that *D*_infr_ < *D*_supr_ again in all subjects, suggesting that interhemispheric transfer is weaker in inferior field. However there is intersubject variation in the relative magnitude of *x*_1_ and *x*_4_: in three subjects *x*_4_ < *x*_1_, with the result that *D*_supr_ > 1, an anomaly that may be tackled by amending the model. In the revised weight allocation for these factors:

(11′) $$x_{4}=US\;\text{and}\;x_{4}^{-1}=BS\;\text{and}\;BI=UI=1$$

The revised weight allocation recognises two separate causes for the display*elevation interaction, both arising from the inferior/superior asymmetry in interhemispheric transfer. One is a deceleration in superior bilateral displays, as before, and the other is an acceleration of unilateral displays with superior target locations (as if such targets are detected faster within the ipsilateral hemisphere, as explained in the main text). The optimisation routine now obtains *x*_1_ < *x*_4_ < 1 in all subjects, such that *D*_infr_ < *D*_supr_ < 1, rectifying the anomaly. Note that, in (11′), *BI* = 1, a change from (6′) that results in a corresponding adjustment to the value obtained for *x*_1_ (the weight of the *B* mode of the display factor) such that *D*_infr_ (equal to *B* × *BI*) is unchanged. Furthermore, if *BI* = 1:

$$BS=\frac{D_{\text{supr}}}{D_{\text{infr}}}\;\text{rearranging}\;(7^{\prime })\;-\text{and}\;\text{hence}$$

$$US=BS^{-1}=\frac{D_{\text{supr}}}{D_{\text{infr}}}\;\text{rearranging}\;(11^{\prime })$$

Meaning that the quotient *D*_infr_/*D*_supr_ can be substituted for *US* in Eqs. (A1) and (A3).

## Appendix C

In the initial definition of crowding parameters:

$$x_{5}=BV=UH\;\text{and}\;BH=UV=1\;(8^{\prime })\;(\text{display}*\text{locus}\;\text{interaction})$$

$$\begin{array}{c} x_{7}=BIV=UIH\;\text{and}\;x_{7}^{-1}=BSV=USH\;\text{and}\\ BIH=UIV=BSH=USV=1 \end{array}\;(9^{\prime })\;(\text{display}*\text{elevation}*\text{locus}\;\text{interaction})$$

(10′) $$\begin{array}{c} C_{\text{supr}}^{H}=UH\times USH=C_{\text{supr}}^{V}=BV\times BSV=x_{5}\times x_{7}^{-1}\;\text{and}\\ C_{\text{infr}}^{H}=UH\times UIH=C_{\text{infr}}^{V}=BV\times BIV=x_{5}\times x_{7}^{-1} \end{array}$$

Thus, *x*_5_ applies to the crowded loci (UNL H-locus and BIL V-locus) as a decelerating factor, with the expectation that *x*_5_ > 1 (Eq. (8′)); the V- and H-loci within the same quadrant experience equivalent crowding (Eq. (10′)), but the respective level in the superior and inferior hemifields is further modulated by the factor *x*_7_ (Eq. (9′)).

In the revised formulation, several of the crowding factors are also governed by the interhemispheric transfer factor, *T*.

(A9) $$C_{\text{infr}}^{V}={\left(C_{\text{infr}}^{H}\right)}^{T_{\text{infr}}}$$

(A10) $$C_{\text{supr}}^{V}={\left(C_{\text{supr}}\right)}^{T_{\text{supr}}}$$

(A11) $$C_{\text{supr}}^{H}={\left(C_{\text{supr}}\right)}^{T_{\text{infr}}/T_{\text{supr}}}$$

To implement Eq. (A9):

(A12) $$x_{5}=UH\;\text{and}\;x_{5}^{T_{\text{infr}}}=BV\;\text{and}\;BH=UV=1$$

(A13) $$x_{7}=UIH\;\text{and}\;x_{7}^{T_{\text{infr}}}=BIV\;\text{and}\;BIH=UIV=BSH=USV=1$$

To implement Eqs. (A10) and (A11):

(A14) $$x_{7}^{-1}=USH\;\text{and}\;x_{5}^{P-T_{\text{infr}}}\times x_{7}^{-P}=BSV;\;[\text{where}\;P=T_{\text{supr}}^{2}/T_{\text{infr}}]$$

And the crowding factors are now:

(A15) $$C_{\text{supr}}={\left(UH\times USH\right)}^{T_{\text{supr}}/T_{\text{infr}}}\;\text{and}\;C_{\text{infr}}=UH\times UIH$$

## Appendix D

The final model is specified by the full set of equations:

$$\begin{array}{lll} UIV=S_{IV} & \mathrm{BIV}=S_{IV}\times \frac{\left(1+T_{\text{infr}}\right)}{2}\times C_{\text{infr}}^{T_{\text{infr}}} & \left[\text{M}1]\;\&\;[\text{M}2\right]\\ \mathrm{UIH}=S_{\mathrm{IH}}\times C_{\text{infr}} & \mathrm{BIH}=S_{\mathrm{IH}}\times \frac{\left(1+T_{\text{infr}}\right)}{2} & \left[\text{M}3]\;\&\;[\text{M}4\right]\\ \mathrm{USV}=S_{\mathrm{SV}}\times \frac{\left(T_{\text{supr}}\_T_{\text{infr}}\right)}{2T_{\text{supr}}} & \mathrm{BSV}=S_{\mathrm{SV}}\times \frac{\left(1+T_{\text{supr}}\right)}{2}\times C_{\text{supr}}^{T_{\text{supr}}} & \left[\text{M}5]\;\&\;[\text{M}6\right]\\ \mathrm{USH}=S_{\mathrm{SH}}\times \frac{\left(T_{\text{supr}}\_T_{\text{infr}}\right)}{2T_{\text{supr}}}\times C_{\text{supr}}^{T_{\text{infr}}/T_{\text{supr}}} & \mathrm{BSH}=S_{\mathrm{SH}}\times \frac{\left(1+T_{\text{supr}}\right)}{2} & \left[\text{M}7]\;\&\;[\text{M}8\right] \end{array}$$

where after averaging across equivalent locations in right and left hemifields: *S*_*IV*_, *S*_*IH*_, *S*_*SV*_, and *S*_*SH*_ are the intrinsic processing speeds at the respective locations: inferior V-locus, inferior H-locus, superior V-locus and superior H-locus. *T*_supr_ and *T*_infr_ are the transfer coefficients for superior and inferior hemifields; *C*_supr_ and *C*_infr_ are the crowding coefficients for superior and inferior hemifields.

Note that [M5] and [M7] express the ‘ipsilateral hemisphere solution’ for superior targets in unilateral displays (see Sections 3.4.5 and 4.5.1).

## Footnotes

1 The control data in the two publications is much alike. A small change in the plotted, 2-item RT (−16 ms) increases unilateral slope from 42.6 ms per item (1989) to 44.7 (1994). A similar but still smaller change (38.9 to 39.4  ms per item) takes place in the bilateral data.

2 Assuming target-absent search is serial and exhaustive. If search is terminated before all items are inspected, the ITI will be a lower estimate (Cousineau & Shiffrin, 2005).

## References

[bib0005] Afraz S.R., Montaser-Kouhsari L., Vaziri-Pashkam M., Moradi F. (2003). Interhemispheric visual interaction in a patient with posterior callosectomy. Neuropsychologia.

[bib0010] Alvarez G.A., Cavanagh P. (2005). Independent resources for attentional tracking in the left and right visual hemifields. Psychological Science.

[bib0015] Awh E., Armstrong K.M., Moore T. (2006). Visual and oculomotor selection: Links, causes and implications for spatial attention. Trends in Cognitive Sciences.

[bib0020] Awh E., Pashler H. (2000). Evidence for split attentional foci. Journal of Experimental Psychology: Human Perception and Performance.

[bib0025] Banich M.T. (1998). The missing link: the role of interhemispheric interaction in attentional processing. Brain and Cognition.

[bib0030] Bashore T.R. (1981). Vocal and manual reaction time estimates of interhemispheric transmission time. Psychological Bulletin.

[bib0035] Beirne R.O., Zlatkova M.B., Anderson R.S. (2005). Changes in human short-wavelength-sensitive and achromatic resolution acuity with retinal eccentricity and meridian. Visual Neuroscience.

[bib0040] Braun C.M., Daigneault S., Dufresne A., Miljours S., Collin I. (1995). Does so-called interhemispheric transfer time depend on attention?. American Journal of Psychology.

[bib0045] Braun C.M., Larocque C., Achim A. (2004). Experimental disentangling of spatial-compatibility and interhemispheric-relay effects in simple reaction time (Poffenberger paradigm). Experimental Brain Research.

[bib0050] Bundesen C., Habekost T., Kyllingsbaek S. (2005). A neural theory of visual attention: Bridging cognition and neurophysiology. Psychological Review.

[bib0055] Carlson T.A., Alvarez G.A., Cavanagh P. (2007). Quadrantic deficit reveals anatomical constraints on selection. Proceedings of the National Academy of Sciences USA.

[bib0060] Carrasco M., Evert D.L., Chang I., Katz S.M. (1995). The eccentricity effect: Target eccentricity affects performance on conjunction searches. Perception and Psychophysics.

[bib0065] Carrasco M., Frieder K.S. (1997). Cortical magnification neutralizes the eccentricity effect in visual search. Vision Research.

[bib0070] Carrasco M., Yeshurun Y. (1998). The contribution of covert attention to the set-size and eccentricity effects in visual search. Journal of Experimental Psychology: Human Perception and Performance.

[bib0075] Cave K.R., Wolfe J.M. (1990). Modeling the role of parallel processing in visual search. Cognitive Psychology.

[bib0080] Chakravarthi R., Cavanagh P. (2009). Bilateral field advantage in visual crowding. Vision Research.

[bib0085] Chun M.M., Wolfe J.M. (1996). Just say no: How are visual searches terminated when there is no target present?. Cognitive Psychology.

[bib0090] Clarke S., Miklossy J. (1990). Occipital cortex in man: Organization of callosal connections, related myelo- and cytoarchitecture, and putative boundaries of functional visual areas. Journal of Comparative Neurology.

[bib0095] Corbetta M (1998). Frontoparietal cortical networks for directing attention and the eye to visual locations: Identical, independent, or overlapping neural systems?. Proceedings of the National Academy of Sciences USA.

[bib0100] Cousineau D., Shiffrin R.M. (2005). Termination of a visual search with large display size effects. Spatial Vision.

[bib0105] Deco G., Pollatos O., Zihl J. (2002). The time course of selective visual attention: Theory and experiments. Vision Research.

[bib0110] Demeter S., Rosene D.L., Van Hoesen G.W. (1990). Fields of origin and pathways of the interhemispheric commissures in the temporal lobe of macaques. Journal of Comparative Neurology.

[bib0115] Demirel S., Robinson R. (2003). Upper vs. lower field asymmetry in SAP and SWAP thresholds: Comparison to psychophysical estimates of ganglion cell density. Investigative Ophthalmology and Visual Science.

[bib0120] Desimone R. (1998). Visual attention mediated by biased competition in extrastriate visual cortex. Philosophical Transactions of the Royal Society of London, B.

[bib0125] Duncan J., Humphreys G.W. (1989). Visual search and stimulus similarity. Psychological Review.

[bib0130] Efron R., Yund E.W. (1996). Spatial nonuniformities in visual search. Brain and Cognition.

[bib0135] Eriksen C.W., St James J.D. (1986). Visual attention within and around the field of focal attention: A zoom lens model. Perception and Psychophysics.

[bib0140] He S., Cavanagh P., Intriligator J. (1996). Attentional resolution and the locus of visual awareness. Nature.

[bib0145] Hemond C.C., Kanwisher N.G., Op de Beeck H.P. (2007). A preference for contralateral stimuli in human object- and face-selective cortex. PLoS One.

[bib0150] Herd S.A., O’Reilly R.C. (2005). Serial visual search from a parallel model. Vision Research.

[bib0155] Ibos G., Duhamel J.R., Ben Hamed S. (2009). The spatial and temporal deployment of voluntary attention across the visual field. PLoS One.

[bib0160] Intriligator J., Cavanagh P. (2001). The spatial resolution of visual attention. Cognitive Psychology.

[bib0165] Jans B., Peters J.C., De Weerd P. (2010). Visual spatial attention to multiple locations at once: The jury is still out. Psychological Review.

[bib0170] Kraft A., Muller N.G., Hagendorf H., Schira M.M., Dick S., Fendrich R.M. (2005). Interactions between task difficulty and hemispheric distribution of attended locations: Implications for the splitting attention debate. Brain Research Cognitive Brain Research.

[bib0175] Kraft A., Pape N., Hagendorf H., Schmidt S., Naito A., Brandt S.A. (2007). What determines sustained visual attention? The impact of distracter positions, task difficulty and visual fields compared. Brain Research.

[bib0180] Luck S.J., Hillyard S.A., Mangun G.R., Gazzaniga M.S. (1989). Independent hemispheric attentional systems mediate visual search in split-brain patients. Nature.

[bib0185] Luck S.J., Hillyard S.A., Mangun G.R., Gazzaniga M.S. (1994). Independent attentional scanning in the separated hemispheres of split-brain patients. Journal of Cognitive Neuroscience.

[bib0190] McCormick P.A., Klein R. (1990). The spatial distribution of attention during covert visual orienting. Acta Psychologica (Amst).

[bib0195] Moore T., Armstrong K.M., Fallah M. (2003). Visuomotor origins of covert spatial attention. Neuron.

[bib0200] Mounts J.R., Gavett B.E. (2004). The role of salience in localized attentional interference. Vision Research.

[bib0205] Muller-Plath G., Pollmann S. (2003). Determining subprocesses of visual feature search with reaction time models. Psychological Research.

[bib0210] Oldfield R.C. (1971). The assessment and analysis of handedness: The Edinburgh inventory. Neuropsychologia.

[bib0215] Pollmann S., Zaidel E., von Cramon D.Y. (2003). The neural basis of the bilateral distribution advantage. Experimental Brain Research.

[bib0220] Posner M.I., Snyder C.R., Davidson B.J. (1980). Attention and the detection of signals. Journal of Experimental Psychology.

[bib0225] Previc F.H. (1996). Attentional and oculomotor influences on visual field anisotropies in visual search performance. Visual Cognition.

[bib0230] Previc F.H., Blume J.L. (1993). Visual search asymmetries in three-dimensional space. Vision Research.

[bib0235] Previc F.H., Naegele P.D. (2001). Target-tilt and vertical-hemifield asymmetries in free-scan search for 3-D targets. Perception and Psychophysics.

[bib0240] Reardon K.M., Kelly J.G., Matthews N. (2009). Bilateral attentional advantage on elementary visual tasks. Vision Research.

[bib0245] Rizzolatti G., Riggio L., Dascola I., Umilta C. (1987). Reorienting attention across the horizontal and vertical meridians: Evidence in favor of a premotor theory of attention. Neuropsychologia.

[bib0250] Robinson D.L., Petersen S.E. (1992). The pulvinar and visual salience. Trends in Neurosciences.

[bib0255] Sample P.A., Irak I., Martinez G.A., Yamagishi N. (1997). Asymmetries in the normal short-wavelength visual field: Implications for short-wavelength automated perimetry. American Journal of Ophthalmology.

[bib0260] Santhouse A.M., Ffytche D.H., Howard R.J., Williams S.C., Rifkin L., Murray R.M. (2002). Functional imaging of the mechanisms underlying the bilateral field advantage. Neuroimage.

[bib0265] Sereno A.B., Kosslyn S.M. (1991). Discrimination within and between hemifields: A new constraint on theories of attention. Neuropsychologia.

[bib0270] Sherman S.M. (2005). Thalamic relays and cortical functioning. Progress in Brain Research.

[bib0275] Shipp S. (2003). The functional logic of cortico-pulvinar connections. Philosophical Transactions of the Royal Society of London. Series B: Biological Sciences.

[bib0280] Shipp S. (2004). The brain circuitry of attention. Trends in Cognitive Sciences.

[bib0285] Snow J.C., Allen H.A., Rafal R.D., Humphreys G.W. (2009). Impaired attentional selection following lesions to human pulvinar: Evidence for homology between human and monkey. Proceedings of the National Academy of Sciences USA.

[bib0290] Tootell R.B., Mendola J.D., Hadjikhani N.K., Liu A.K., Dale A.M. (1998). The representation of the ipsilateral visual field in human cerebral cortex. Proceedings of the National Academy of Sciences of the USA.

[bib0295] Treisman A., Sato S. (1990). Conjunction search revisited. Journal of Experimental Psychology: Human Perception and Performance.

[bib0300] Van Essen D.C., Newsome W.T., Bixby J.L. (1982). The pattern of interhemispheric connections and its relationship to extrastriate visual areas in the macaque monkey. Journal of Neuroscience.

[bib0305] Van Essen D.C., Zeki S.M. (1978). The topographic organization of rhesus monkey prestriate cortex. Journal of Physiology.

[bib0310] Wolfe J.M. (1994). Guided Search 2.0: A revised model of visual search. Psychonomic Bulletin & Review.

[bib0315] Wolfe J.M. (1998). What can 1 million trials tell us about visual search?. Psychological Science.

[bib0320] Wolfe J.M., O’Neill P., Bennett S.C. (1998). Why are there eccentricity effects in visual search? Visual and attentional hypotheses. Perception and Psychophysics.

[bib0325] Woodman G.F., Luck S.J. (2003). Serial deployment of attention during visual search. Journal of Experimental Psychology: Human Perception and Performance.

[bib0330] Yund E.W., Efron R., Nichols D.R. (1990). Detectability as a function of spatial location: Effects of selective attention. Brain and Cognition.

[bib0335] Yund E.W., Efron R., Nichols D.R. (1990). Detectability gradients as a function of target location. Brain and Cognition.

